# *Bacteroides fragilis* strain ZY-312 facilitates colonic mucosa regeneration in colitis *via* motivating STAT3 signaling pathway induced by IL-22 from ILC3 secretion

**DOI:** 10.3389/fimmu.2023.1156762

**Published:** 2023-04-11

**Authors:** Wendi Zhang, Qian Zhou, Hongbin Liu, Jiahui Xu, Ruo Huang, Binhai Shen, Yandong Guo, Xiuyun Ai, Jun Xu, Xinmei Zhao, Yangyang Liu, Ye Wang, Fachao Zhi

**Affiliations:** ^1^ Guangdong Provincial Key Laboratory of Gastroenterology, Institute of Gastroenterology of Guangdong Province, Department of Gastroenterology, Nanfang Hospital, Southern Medical University, Guangzhou, China; ^2^ Department of Gastroenterology, The Second Affiliated Hospital of Guangzhou Medical University, Guangzhou, China; ^3^ Huiqiao Medical Center, Nanfang Hospital, Southern Medical University, Guangzhou, China; ^4^ Guangzhou ZhiYi Biotechnology Co., Ltd., Guangzhou, China

**Keywords:** inflammatory bowel disease, *Bacteroides fragilis* strain ZY-312, colonic mucosa regeneration, Stat3 signaling pathway, ILC3, IL-22

## Abstract

**Introduction:**

Probiotics play critical roles in relieving inflammatory bowel disease (IBD). However, the underlying mechanism of *Bacteroides fragilis* strain ZY-312 (*B. fragilis*) for colonic mucosa regeneration in IBD remains unclear.

**Methods:**

The weight loss, disease activity index (DAI), colon length, and histopathology-associated index (HAI) were evaluated the therapeutic effects of *B. fragilis* in a DSS-induced colitis mouse model. Colonic mucosa proliferation and apoptosis level, and mucus density were detected by histological stain. Gut microbiota was sequenced by 16srRNA analysis. The expression of signal transducer and activator of transcription 3 (STAT3) phosphorylation in colonic mucosa was detected in *B. fragilis*-treated mice in colitis. *B. fragilis*-regulated immunity factors of motivating downstream STAT3 phosphorylation were screened by ELISA and flow cytometry. Lastly, *B. fragilis*-mediated colonic mucosa regeneration effects were verified though the knockout of STAT3 (*Stat3* ^△IEC^) and IL-22 (IL-22^-/-^) in mice, and inhibitor of STAT3 and IL-22 in co-culture model.

**Results:**

*B. fragilis* alleviated DSS-induced colitis in mice with less weight loss, DAI, colon length shortening, and HAI. Further the results showed that *B. fragilis* motivated STAT3 phosphorylation in colonic mucosa with the upregulation of proliferation index Ki-67 and mucus density, the downregulation of apoptosis level, and the modulation of gut microbiota through a *Stat3* ^△IEC^ mice model and STAT3 inhibitor-added model in vitro. Meanhwhile we found that *B. fragilis* promoted IL-22 production, and increased the percentage of IL-22-secreting type 3 innate lymphocytes (ILC3) in colitis. Consequently, We identified that *B. fragilis* did not increase the expression of pSTAT3, either proliferation level, mucus density, or alter gut microbiota in *IL-22* ^-/-^ mice.

**Discussion:**

*B. fragilis* may indirectly motivate ILC3 to secrete IL-22, followed by IL-22-induced STAT3 phosphorylation, hence promoting colonic mucosa regeneration in colitis. It indicates that *B. fragilis* has the potential to be a biological agent for IBD therapy.

## 1 Introduction

Inflammatory bowel disease (IBD) is an autoimmune disorder that includes Ulcerative colitis (UC) and Crohn’s disease (CD). Environmental exposures in genetically susceptible individuals cause an imbalance in the intestinal microbiota and intestinal barrier. Consequently, the intestinal immune system triggers persistent inflammation, injuring the intestinal mucosa (1). Hence, The imbalance of Genetic and environmental factors, immunity, and intestinal flora will contribute to IBD. A recent study reports that endoscopic mucosal repair is an important indicator for evaluating colitis remission (2). It suggests that facilitating intestinal mucosa regeneration is a key strategy for IBD relief.

Intestinal mucosa is a crucial modulator of intestinal homeostasis that segregates commensal microorganisms in the intestinal lumen and the host immune system under the intestinal mucosa layer (3). Meanwhile, it enables to establish an immunological environment permissive to colonization by commensal bacteria (4). Importantly, goblet cells, one of the different types of intestinal epithelial cells, exert a vital role in the colonization of commensal bacteria and the rejection of pathogenic bacteria through secreting mucus to attach to the surface of the intestinal mucosa (5). A study reported that the intestinal goblet cell density was lower in germ-free (GF) mice which were different from conventionally raised mice (6). And bacterial products including lipopolysaccharides and peptidoglycans could stimulate mucus secretion and restore mucus thickness in GF mice to a similar extent as in conventionally raised mice (7). It suggests that the gut microbiota is important for the formation of the mucus layer. Also, there are a series of mucus-degrading probiotics, including *Akkermansia muciniphila* and *Bacteroides fragilis*, which perform protective effects in colitis by acting on mucus (8). However, the underlying mechanism of *Bacteroides fragilis* on intestinal mucus secretion and gut microbiota remains unclear. It has been reported that the non-toxigenic strain *Bacteroides fragilis-*derived polysaccharide A could activate CD4+ T cells to secrete Interleukin (IL) -10, hence protecting against CD-like colitis (9). IL-10 has anti-inflammatory effects in colitis by motivating IL-10 receptors (IL-10R) including IL-10R1 and IL-10R2 (10). However, IL-22, belonging to the IL-10 inflammatory family, has two receptors including IL-22RA1 and IL-10R2, and shares common receptor with IL-10 (11). Whether the non-toxigenic strain *Bacteroides fragilis* depends on IL-22-mediated IL-10R2 activation to perform anti-inflammatory effects in colitis remains unclear. In addition, IL-22 mainly derives from type 3 innate lymphocytes (ILC3) (12) and helper T (Th) 17 cells (13), and it could motivate classic signal transducer and activator of transcription 3 (STAT3) signaling pathway to exert proliferation effects in the intestinal mucosa (14). IL-22 could also promote symbiotic bacteria colonization through colonic mucosa saccharification (15). Moreover, several interleukins including IL-22 have been shown to regulate goblet cell differentiation and mucus secretion (16). It suggests that immunity-mediated IL-22 secretion has regulating effects in intestinal mucosa regeneration, and intestinal microbiota. Whether *Bacteroides fragilis* mediates IL-22 signaling to promote intestinal mucosa regeneration and modulate intestinal microbiota maintains unknown.

*Bacteroides fragilis* strain ZY-312 (*B. fragilis*), belonging to *Bacteroides*, was previously isolated from the feces of a healthy infant by our team (17, 18). O’Toole et al. proposed that *B. fragilis* is likely to become a second-generation probiotic with biological applications (19). *B. fragilis* mainly exists in the small intestine and colon through a metabolic engineering approach (20). We found that *B. fragilis* promoted colon tissue proliferation in an antibiotic-related diarrhea rat model (21). Additionally, *B. fragilis* has the function of promoting mucus secretion, regulating immunity, and modulating intestinal flora to relieve Cronobacter sakazakii-induced neonatal necrotizing enterocolitis in neonatal rats (22). It suggests that *B. fragilis* has influences on epithelium especially goblet cell regeneration, immune homeostasis, and modulating intestinal flora. However, The effects and underlying mechanism of *B. fragilis* for intestinal mucosa regeneration, immunity regulation, and intestinal flora in dextran sodium sulfate (DSS)-induced UC-like colitis remains unclear.

In our work, we verified that *B. fragilis* promoted IL-22 production in colon tissue in DSS-induced colitis. Hence we assumed that *B. fragilis* may motivate the IL-22 signaling pathway to promote intestinal mucosa regeneration and modulate intestinal flora in colitis. To verify the assumption, we administered *B. fragilis* to mice in a DSS-induced colitis mouse model and found that *B. fragilis* facilitates colonic mucosa proliferation, and mucus secretion, and alters gut microbiota in colitis *via* motivating the STAT3 pathway induced by IL-22 from ILC3 secretion. Given the biological therapeutic effects of *B. fragilis*, it indicates that *B. fragilis* has the potential to be a biological agent for IBD therapy.

## 2 Methods

### 2.1 Establishment of dextran sulfate sodium-induced experimental colitis model

The mouse model of DSS-induced experimental colitis was constructed by previously described methods (23). C57BL/6, STAT3 conditional gene-knockout mice (*Stat3* ^△IEC^) and IL-22 gene-knockout mice (*IL-22* ^-/-^) mice were gavaged with or without *B. fragilis* ZY-312 (1×10^9^ CFU per mouse) for 14 days during the pretreatment stage, followed by the induction of colitis with 2.5% DSS (MP Biomedicals, Santa Ana, CA, USA; #9011-18-1) for 5 days. Mice were then sacrificed 3 days after DSS withdrew. The mouse model of DSS-induced colitis was evaluated for the DAI (**Table S1**), weight loss score, fecal traits, and blood in stools. The HAI (**Supplementary Table S2**) was determined to assess colon inflammation. All experiments were performed using 6- to 8-week-old sex-matched mice. C57BL/6 mice were purchased from SPF (Beijing) Biotechnology Co., Ltd. (Beijing, China). *Stat3* ^△IEC^ and *IL-22* ^-/-^ mice were purchased from GemPharmatech Co., Ltd. (Jiangsu, China). Cre-negative littermates were used as wild-type (WT) controls. The generation and validation of conditional knockout STAT3 alleles and IL-22 knockout are described in the **Supplementary Table S5** and **Supplementary methods**. All mice were bred and maintained under specific pathogen-free conditions.

### 2.2 Culture of *Bacteroides fragilis* strain ZY-312

*Bacteroides fragilis* strain ZY-312 (*B. fragilis*) was cultured in 5% fetal bovine serum, 9.5 mL of tryptic soy broth, and 100 µL of passaging solution at 37°C for 24 h under anaerobic conditions.

### 2.3 Isolation and detection of colonic lamina propria immune cells

The colon was isolated and cut longitudinally and divided into segments of 0.5 cm in length. Then the colon was cleaned twice and placed in 20 ml digestive fluid (5 mM EDTA, 100× Hepes, 5% FBS, HBSS without calcium and magnesium) for 20 min twice at 37°C. The rest of the colon was digested using the Lamina Propria Dissociation Kit (Miltenyi Biotec, Bergisch Gladbach, Germany) according to the manufacturer’s protocols. Next, CLP immune cells were collected and further purified by density gradient centrifugation with 40% and 80% Percoll–RPMI solution. Single CLP immune cells were collected in the interphase. These cells were stained with the indicated antibodies (**Table S3**) for detection according to a flow cytometry gating strategy (**Figure S6**). Results were read using a Flow Cytometer (Aria III; BD Bioscience, Franklin Lakes, NJ, USA). Data were analyzed using FlowJo 10.0.

### 2.4 Colonic crypt isolation and colonic organoid co-culture construction

Colonic crypts were obtained from colons of 4-week-old C57BL/6 mice. As previously described (24), the colon was dissected and washed with phosphate-buffered saline (PBS) and then chopped into pieces. After washing 20 times with PBS, the pieces were incubated in Gentle Cell Dissociation Reagent (Stem Cell) for 30 min at 25°C. The mixture was filtered through a 70 μm cell strainer, crypt fractions were isolated and washed with DMEM/F12, and purified colonic crypt fractions were acquired by centrifugation. Next, the colonic crypt was mixed with Matrigel (Corning) and evaluated using the IntestiCult OGM Mouse Kit (Stem cell, Canada) with 50 µl of the mixture added to 24-well plates. Organoid counting was performed according to the manufacturer’s protocols. For the co-culture system containing CLP and colonic organoids, CLP were isolated as described above. Next, the isolated CLP were cultured with colonic organoids at a ratio of 7:1 in Matrigel. *B. fragilis* (10^4^ CFU/well) and murine TNF-α (60 ng/mL; Peprotech, Rocky Hill, NJ, USA) were added independently or simultaneously to the culture medium for 24 h. Murine IL-22 (5 ng/ml, Peprotech), anti-IL-22 neutralizing antibody (0.1 μg/mL, AF582; RD, Minneapolis, MN, USA) (25), and Stattic (15 μM; Selleck, Houston, TX, USA) (26) were also added to organoids for 24 hours. The development of colonic organoids from day 1 to day 6 and the co-culture model is described (**Figure S1**).

### 2.5 Histological analysis

The mouse colon tissue was fixed in 4% paraformaldehyde for 24 h, embedded in paraffin, and cut into sections at a thickness of 4 µm. For immunohistochemical staining, the colonic sections were subjected to deparaffinization, hydration, antigen retrieval, quenching of endogenous peroxidase, and blocking procedures. All slices were then incubated with the primary antibodies against pSTAT3 and Ki-67 (**Figure S2**) at 4°C overnight followed by incubation with biotinylated secondary antibodies for 30 min and visualization using a 3,3′-Diaminobenzidine Kit (ZSGB-BIO, Beijing, China). For immunofluorescence staining for the detection of pSTAT3, PCNA, and cleaved Caspase-3, the colonic sections were processed by the methods described above. A terminal deoxynucleotidyl transferase dUTP nick end labeling (TUNEL) assay was performed using the Servicebio Fluorescein (FITC) TUNEL Cell Apoptosis Detection Kit (Wuhan, China) following the manufacturer’s protocols. Alcian blue and Periodic Acid-Schiff (PAS) staining (Biossci, China) was performed following the manufacturer’s protocols.

### 2.6 Colonic epithelial cell isolation

The colon epithelium was isolated according to the previously reported method. In brief, colon tissues were washed thoroughly in cold PBS. Colons were then cut into 5 mm pieces, added into a digestion buffer (5 mM EDTA and 2 mM DTT in Hanks balanced salt solution; Sigma, St. Louis, MO, USA), and incubated at 37°C for 30 min on a rotating platform. Equal volumes of HBSS were added to stop digestion and the solution was filtered through a 70 μm cell strainer. The filtered solution was centrifuged at 4°C and 400g for 10 min to obtain colonic epithelial cell pellets for protein isolation.

### 2.7 Western blot

Western blotting was performed as described previously (27).The proteins were separated by SDS–PAGE and analysed by immunoblotting with rabbit polyclonal antiserum to pSTAT3 (CST #9145, Danvers, MA, USA), STAT3 (CST #4904), IL-22 (bs-2623R), proliferating cell nuclear antigen (PCNA) (CST #13110), Caspase-3 (CPP32 4-1-18), cleaved Caspase-3 (CST #9661), P38 (CST #9212), pP38 (CST #4511), ERK (CST #4695), pERK (CST#4370), GAPDH (CST #5174) antibodies were used to measure colonic epithelial protein expression.

### 2.8 Quantibody array detection

Colon samples were collected and analyzed using the Quantibody^®^ Mouse Inflammation Array 1 (QAM-INF-1) involving 40 cytokines (**Table S4**) according to protocols in the user manual. Fluorescence signals were detected using Axon GenePix, and the relative levels of cytokines were determined. Differentially expressed genes (DEGs) were obtained using FDR < 0.05 and fold change (FC) > 2 as thresholds. A Gene Ontology (GO) enrichment analysis of DEGs was implemented using the topGO R package, correcting for gene length bias. The ClusterProfiler R package was used to evaluate DEG enrichment for Kyoto Encyclopedia of Genes and Genomes analysis (KEGG) pathways. The fisher exact test was used, and data packets were clusterProfiler from R/Bioconductor. The selection criteria were that the number of different proteins on a certain term/Pathway or GO was >=5, and p_value <0.05. term/Pathway or GO was obtained in descending order according to the value of Count, and the first 12 results were taken. The definition of enrich_factor = (the number of differential genes in a term (that is, Count)/the total number of differential genes in the database term/the total number of genes in the database). A protein-protein interaction (PPI) network of upregulated DEGs was constructed using the String database.

### 2.9 RNA isolation and real-time PCR

RNA was isolated using a column-based isolation kit (EZbioscience, Roseville, MN, USA) according to the manufacturer’s instructions, and concentrations were measured using a spectrophotometer (NanoDrop 2000, Thermo Fisher, Waltham, MA, USA). Equal amounts of RNA (1 μg) were used to generate cDNA by reverse transcription and real-time PCR was performed to measure gene expression levels. In brief, 2μg of diluted extracted RNA was converted to cDNA, then validated primers (**Table S6**) and SYBR Green (Takara, Japan) were added to the cDNA and the mix was run in LightCycler480 (Roche, USA). *P*<0.05 was considered significant.

### 2.10 Enzyme-linked immunosorbent assay

Colon and blood samples were centrifuged at 4°C and 2500 rpm for 20 min and serum was collected. Colon samples were homogenized in lysis buffer (RIPA with protease inhibitor; Beyotime, Shanghai, China) and whole protein concentrations were measured by a bicinchoninic acid assay. Inflammatory factor (IL-22, IL-1β, IL-6, IL-23, IL-10, TNF-α, IFN-γ, and IL-17) Enzyme-linked Immunosorbent Assay (ELISA) kits (Thermo Fisher Scientific) (**Table S3**) were used to measure inflammatory factor concentrations according to the manufacturer’s protocols. Results were read by a microplate reader (SpectraMax190; Molecular Devices, Sunnyvale, CA, USA), and concentrations were calculated using SoftMax Pro 5.0. IL-22 blockage was performed by IL-22 capture antibody incubation overnight at 4°C.

### 2.11 *In vitro* cell function assay

Mouse colon cancer cells MC38 were cultured in DMEM (Gibco, USA) supplemented with 10% serum (Hyclone, Logan, UT, USA) for routine culture and in DMEM with 0.5% serum for cell function experiments. To evaluate the cell proliferative capacity, cells were seeded in a 96-well-plate at a density of 3000 cells/well and a Cell Counting Kit-8 (CCK-8; Dojindo, Kumamoto, Japan) assay was performed. OD_450_ values were measured. A cell cycle analysis was performed by *in vitro* propidium iodide (PI) incorporation following the manufacturer’s instructions (Dojindo). PI-incorporated cells were measured by flow cytometry. Data were analyzed using FlowJo 10.0.

### 2.12 16srRNA analysis

The colonic fecal microbiota composition in mice was determined by 16S rRNA gene amplification. Briefly, a magnetic bead extraction kit (Qiagen, Valencia, California, USA) was used to extract genomic DNA from feces. DNA concentration and integrity were measured by agarose gel electrophoresis. The 16S rRNA gene V3–V4 region was amplified from the genomic DNA in a 25 µl reaction using the universal bacterial primers: (341F, 5' - CCTAYGGGRBGCASCAG - 3', and 806R, 5' - GGACTACNNGGGTATCTAAT - 3'). The PCR products were detected through agarose gel electrophoresis and purified with Universal DNA Purification and Recovery Kit (TianGen, China, Catalog #: DP214). Sequencing was performed on an Illumina NovaSeq 6000 with two paired-end read cycles of 250 bases each (IlluminaInc., San Diego, CA, United States). The two paired FASTQ files were base called from the Illumina raw sequence read data and the quality of the raw sequence reads was assessed using Qiime V1.9.1. After trimming, operational taxonomic units (OTUs) were generated by using Uparse v7.0.1001 with a 97% similarity cutoff. The representative read of each OTU was selected by using the Quantitative Insights into Microbial Ecology (QIIME) package. All representative reads were annotated and blasted against the Silva database using the RDP classifier (confidence threshold was 80%). The microbial richness and diversity in fecal content samples were estimated using the alpha diversity that includes the Shannon index. The UniFrac distance matrix performed by QIIME software was used for the unweighted UniFrac Principal coordinates analysis (PCoA), and phylogenetic tree construction. Linear discriminant analysis effect size (LEfSe) based linear discriminant analysis (LDA) and cladogram were generated to assess differentially abundant microbial taxa. The 16S rRNA gene amplicon sequencing and analysis were conducted by Novogene Bioinformatics Technology Co., Ltd (Beijing, China).

### 2.13 Statistical analysis

Statistical analyses were performed using GraphPad Prism 8.0 (GraphPad Software). All variables are expressed as Means ± SEM, as noted in the figure legends. A Student’s t-test was used forcomparisons between two groups. One-way ANOVA and Tukey’stests were used for comparisons among three or more groups, and Two-way ANOVA was used tocompare body weight and DAI. While non-parametric data were analyzed with a Mann–Whitney U-test. P < 0.05 was the threshold for significance.

## 3 Results

### 3.1 *B. fragilis* alleviated DSS-induced colitis by promoting colonic mucosa proliferation and mucus secretion, and modulating microbiota

**Figure 1:**
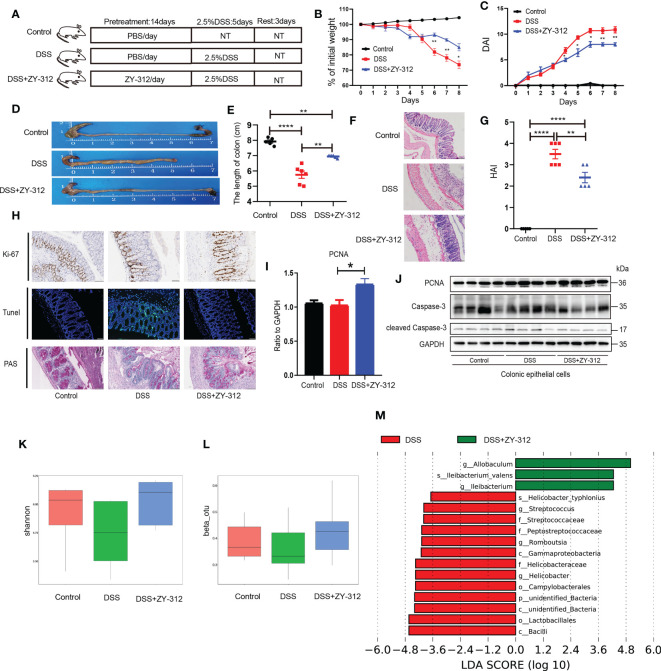
*B. fragilis* alleviated DSS-induced colitis by promoting colonic mucosa proliferation and mucus secretion, inhibiting apoptosis. **(A)** The route of the DSS-induced experimental colitis mouse model was as follows, mice were pretreated with *B. fragilis* strain ZY-312 (1x10^9^ CFU) (DSS+ZY-312 group) or phosphate buffer (PBS) (DSS group) for 14 days orally. Next mice were treated with 2.5% DSS for 5 days, then sacrificed 3 days after DSS withdrew, Control group (N=5), DSS group (N=6), and DSS+ZY-312 group (N=5). N, number of mice, NT means no treatment. **(B, C)** Percent of weight loss **(B)** and disease activity index (DAI) **(C)** were monitored daily starting from DSS administration. **(D)** Representative of colon morphology. **(E)** The colon length of mice. **(F)** H&E staining of colon tissues. Scale bars, 200 μm. **(G)** The histopathological associated index (HAI). **(H)** Immunochemistry analysis of the level of Ki-67 (upper), Immunofluorescence analysis of Tunel (middle), PAS staining of mucus in colon sections (below). Scale bars, 200 μm. **(I)** Quantitative PCR (qPCR) analysis of PCNA in colon tissue. **(J)** Western blot analysis of PCNA and cleaved Caspase-3 in colonic epithelial cells. **(K, L)** The α **(K)** and β **(L)** diversity analysis of microbiota between groups. **(M)** LEfSe analysis of differential bacteria in feces between the DSS group and ZY-312 group with different levels meeting a significant LDA threshold value of >2.5. Data are presented as Mean ± SEM **(B, C, E, G, I, K)**. Statistical analysis was performed using two-way ANOVA **(B, C)**, and one-way ANOVA **(E, G)**. *p < 0.05,**p < 0.01, ****p < 0.001.

To evaluate whether *B. fragilis* has a protective role in DSS-induced colitis, we constructed a DSS-induced colitis mouse model and administered *B. fragilis* orally (**Figure 1A**). Compared with the DSS group, mice receiving *B. fragilis* (DSS+ZY-312 group) had less weight loss (**Figure 1B**), disease activity index (DAI) (**Figure 1C**), colonic constriction (**Figures 1D, E**), and HAI (**Figures 1F, G**). Further, we found that *B. fragilis* increased the level of proliferation index Ki-67 and PCNA, promoted mucus expression, and decreased the level of cleaved Caspase-3 and TUNEL-positive cells compared to the DSS group (**Figures 1H–J**). And flow cytometry results showed that *B. fragilis* promoted colonic epithelium cell cycle progression compared with the DSS group (**Figure S1E**). For further validation *in vitro*, we constructed a co-culture model that included colonic organoids and CLP-derived immune cells to imitate the colonic anatomical structure in mice (**Figures S1A, B**). And we found that *B. fragilis* increased the PCNA level and decreased the cleaved Caspase-3 level in colonic organoids from the co-culture model (**Figure S1D**).

Based on that mucin glycans in the mucus layer could provide the energy source for intestinal microbiota (28), we attempted to investigate the influence of *B. fragilis* on intestinal microbiota. The results showed that *B. fragilis* elevated α, and β diversity compared to the DSS group (**Figures 1K, L**). The LEfSe results showed that the abundance of pathogenic bacteria including *Gammaproteobacteria*, *Helicobacteraceae*, *Helicobacter_typhlonlus*, *Streptococcus*, *Peptostreptococcaceae*, *Campylobacterales* were increased in the DSS group. While *B. fragilis* increased the abundance of *Allobaculum* (**Figure 1M**). *Allobaculum*, belonging to the erysipelaceae class, physiologically can use carbohydrates to produce butyric acid and lactate to exert an anti-inflammatory effect (29). It indicated that *B. fragilis* alleviates DSS-induced colitis by promoting colonic mucosa proliferation, and mucus secretion, and modulating microbiota.

### 3.2 *B. fragilis* motivated STAT3 phosphorylation to facilitate colonic mucosa proliferation and mucus secretion, and alter gut microbiota

**Figure 2:**
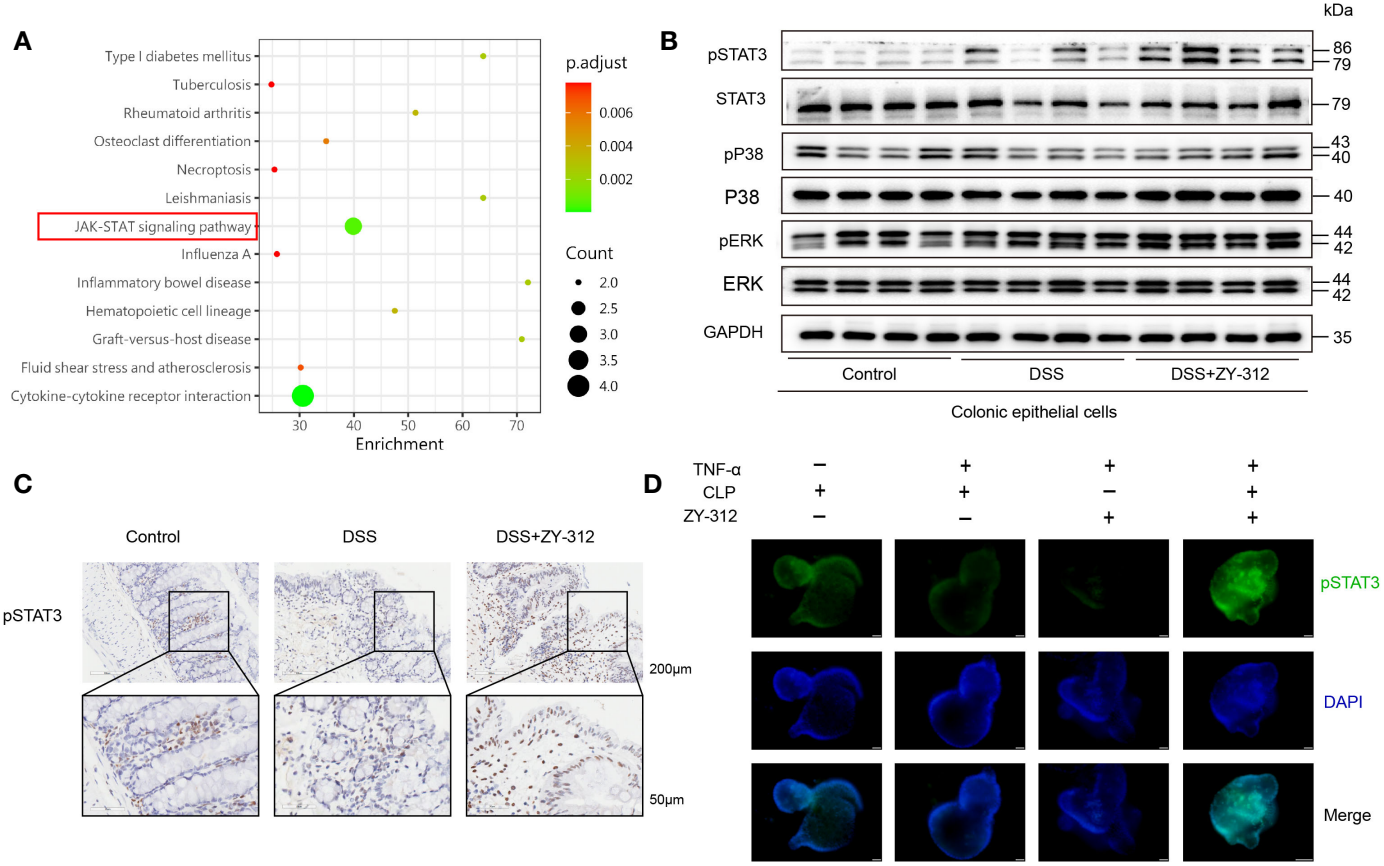
*B fragilis* motivated STAT3 phosphorylation in colonic mucosa. **(A)** KEGG analysis (DSS+ZY-312 group versus DSS group) of gene functions from colon tissue in DSS-induced colitis, linking *B. fragilis* with higher relation with the JAK-STAT signaling pathway in colitis (marked by a red box). **(B)** Western Blot analysis of pSTAT3/STAT3, pP38/P38, and pERK/ERK signaling pathway in colonic mucosa. **(C)** Immunohistochemistry analysis of pSTAT3 in colonic mucosa. Scale bars, 200 μm (upper), 50 μm (below). **(D)** Immunofluorescence analysis of pSTAT3 in colonic organoids from co-culture model, The co-culture model was added with (+) or without (-) *B. fragilis* strain ZY-312 (10^4^ CFU/well) after TNF-α (60ng/ml) induced inflammation for 24 hours *in vitro*. (Scale bars,50 μm).

The proliferation pathways, including STAT3 (30), p38 (31), and extracellular-signal-regulated kinase (ERK) (32), participate in intestinal mucosa proliferation and apoptosis in IBD. Next, we attempted to screen and verify which signaling pathway was involved in *B. fragilis-*mediated colonic mucosa proliferation. Through the protein microarray of colon tissue, the KEGG analysis showed that *B. fragilis* participates in the Janus kinase (JAK)-STAT signaling pathway (**Figure 2A**). And western blotting (WB) and immunohistochemical results showed that *B. fragilis* elevated pSTAT3 level in the colonic mucosa compared to the DSS group (**Figures 2B, C**). Besides, immunofluorescence results demonstrated that *B. fragilis* increased pSTAT3 expression in the colonic organoids (**Figure 2D**). It suggests that *B. fragilis* elevates the pSTAT3 level in the colonic mucosa.

**Figure 3:**
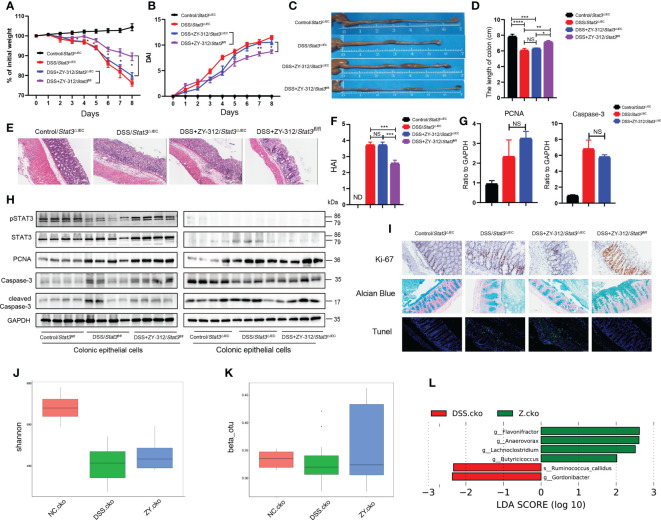
*B. fragilis* motivated STAT3 phosphorylation in colonic mucosa to promote colonic enterocytes and goblet cell proliferation, inhibit apoptosis, and alter gut microbiota. **(A, B)** Percent of weight loss **(A)** and DAI **(B)** were monitored daily starting from DSS administration. Control/*Stat3* ^△IEC^ group (N=4), DSS/*Stat3* ^△IEC^ group (N=7), DSS+ZY-312/*Stat3* ^△IEC^ group (N=7), DSS+ZY-312/*Stat3* ^fl/fl^ group (N=7). N, number of mice. **(C)** Representative colon morphology. **(D)** Statistical analysis of colon length. **(E)** H&E staining of colon tissues. (Scale bars, 200 μm). **(F)** Statistical analysis of HAI. **(G)** qPCR analysis of PCNA and Caspase-3 in colon tissue. **(H)** Western Blot analysis of pSTAT3, STAT3, PCNA, cleaved Caspase-3, Caspase-3 in colonic epithelial cells. **(I)** Immunochemistry analysis of Ki-67 (upper), Alcian blue staining of mucus (middle), Tunel assay analysis (below) of colon tissue. (Scale bars, 200 μm). **(J, K)** The α**(J)** and β**(K)** diversity analysis of microbiota in *Stat3* ^△IEC^ mice between groups. **(L)** LEfSe analysis of differential bacteria in feces between the DSS group and ZY-312 group with different levels meeting a significant LDA threshold value of >2 in *Stat3* ^△IEC^ mice. NC.cko indicates Control/*Stat3* ^△IEC^ group. DSS.cko indicates DSS/*Stat3* ^△IEC^ group. Z.cko indicates DSS+ZY-312/*Stat3* ^△IEC^ group. Data are presented as Mean ± SEM (A, B, D, F, G). Statistical analysis was performed using two-way ANOVA **(A, B)**, and one-way ANOVA **(D, F, G)**. *p < 0.05,**p < 0.01, ***p < 0.005, ****p < 0.001. ND indicates undetectable. NS indicates no significance.

Next, we constructed a DSS-induced colitis model and administered *B. fragilis* orally in *Stat3* ^△IEC^ mice (**Figure S2**). The results showed that *Stat3* ^△IEC^ mice receiving *B. fragilis* were more susceptible to DSS-induced colitis than the wild-type (*Stat3* ^fl/fl^) mice receiving *B. fragilis*, evidenced by more weight loss (**Figure 3A**), a higher DAI score (**Figure 3B**), a shorter colon length (**Figures 3C, D**), and a higher HAI (**Figures 3E, F**; **Figure S3**). To investigate whether *B. fragilis* motivated STAT3 phosphorylation to regulate colonic mucosa proliferation and mucus secretion, and modulate microbiota. The qPCR, WB, immunohistochemistry, and immunofluorescence results showed that *B. fragilis* did not increase the PCNA, Ki-67 levels, or mucus density, either decrease the cleaved Caspase-3 level or TUNEL-positive cells density in the *Stat3* ^△IEC^ mice contrast to the wild-type mice (**Figures 3G–I**; **Figure S3**). *In vitro* assay, we inhibited STAT3 expression through a STAT3 pathway inhibitor in the colonic organoids and MC38 co-culture model (**Figure S1C**). The immunofluorescence results showed that *B. fragilis* did not elevate PCNA expression, or decrease the cleaved Caspase-3 level in the colonic organoids. And CCK8 results showed that *B. fragilis* did not increase cell viability, or decrease cleaved Caspase-3 level once inhibiting STAT3 in MC38 (**Figure S4A–C**). In addition, The α, β diversity and LEfSe analysis showed a similar microbiota composition between the DSS+ZY-312 group and the DSS group in *Stat3* ^△IEC^ mice (**Figures 3J–L**). Together, these results suggest that *B. fragilis* facilitated colonic mucosa proliferation and mucus secretion, and altered gut microbiota through the STAT3 signaling pathway.

### 3.3 *B. fragilis* up-regulated IL-22, motivating STAT3 phosphorylation in the colonic mucosa

**Figure 4:**
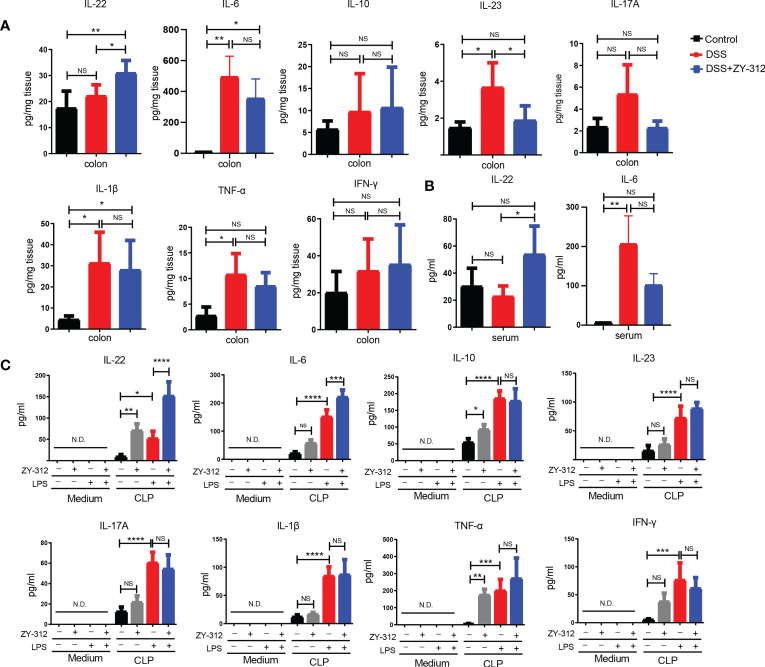
*B.fragilis* promoted IL-22 production, but not IL-1β and TNF-α mediated classical proinflammatory pathway in DSS-induced colitis. **(A)** ELISA analysis of the inflammatory factors including IL-22, IL-6, IL-23, IL-1β, TNF-α, IFN-γ, IL-17A, and IL-10 from colon tissue in DSS-induced colitis. **(B)** ELISA analysis of IL-22, and IL-6 from serum in DSS-induced colitis. **(C)** ELISA analysis of IL-22, IL-6, IL-23, IL-1β, TNF-α, IFN-γ, IL-17A, and IL-10 in supernatant from co-culture model including *B. fragilis* strain ZY-312 and CLP after lipopolysaccharide (LPS,1ug/ml) stimulation for 12 hours *in vitro*. Data are presented as Mean ± SEM **(A–C)**. Statistical analysis was performed using one-way ANOVA. *p < 0.05,**p < 0.01, ***p < 0.005, ****p < 0.001.ND indicates undetectable. NS indicates no significance.

Inflammatory cytokines, such as IL-22, IL-10, and IL-6, could activate STAT3 phosphorylation to exert proliferation effects (33). Hence, we detected the level of related inflammatory factors. The ELISA results showed that *B. fragilis* promoted IL-22 production in the colon and serum (**Figures 4A, B**), and decreased the level of IL-23, while *B. fragilis* has no effect on IL-6, IL-10, IL-17A, IL-1β, tumor necrosis factor-alpha (TNF-α), and interferon-gamma (IFN-γ) compared to the DSS group. IL-22 is mainly produced by CLP-derived immune cells in the colon (34). Therefore, we dissociated CLP-derived immune cells and treated them with *B. fragilis* to detect the level of IL-22 in the culture medium (supernatant) *in vitro*. The ELISA results showed that *B. fragilis* promoted CLP-derived immune cells to secrete IL-22 and IL-6, but did not influence IL-10, IL-23, IL-17A, IL-1β, TNF-αor IFN-γ (**Figure 4C**). Meanwhile, the flow cytometry results showed that the supernatant from *B. fragilis-*treated with CLP-derived immune cells promoted cell cycle progression of MC38 (**Figure S7A**).

**Figure 5:**
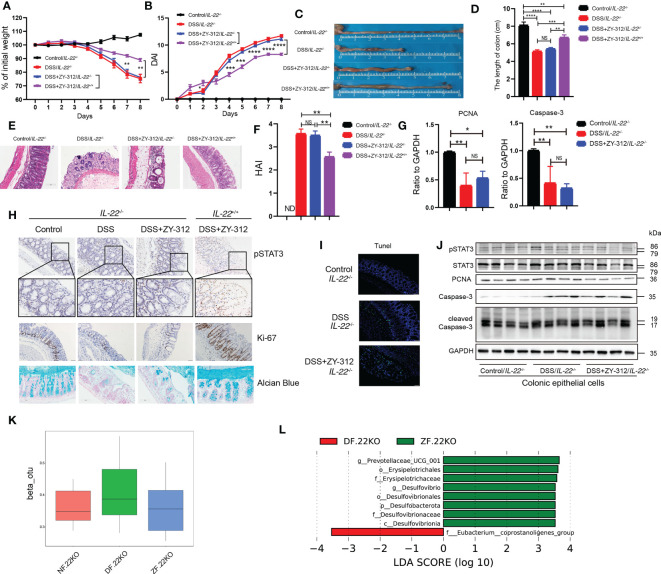
*B. fragilis* up-regulated IL-22/pSTAT3 pathway to facilitate colonic enterocytes proliferation and mucus secretion, inhibit apoptosis, and impact gut microbiota. **(A, B)** Percent of weight loss **(A)** and DAI **(B)** were monitored daily starting from DSS administration. Control/*IL-22^-/-^* group (N=4), DSS/*IL-22^-/-^* group (N=7), DSS+ZY-312/*IL-22^-/-^* group (N=8), DSS+ZY-312/*IL-22^+/+^* group (N=7). N, number of mice. **(C)** Representative colon morphology. **(D)** Statistical analysis of colon length. **(E)** H&E staining of colon tissues (Scale bars, 200 μm). **(F)** Statistical analysis of HAI. **(G)** qPCR analysis of Caspase-3 and PCNA in colon tissue. **(H)** Immunochemistry analysis of pSTAT3 and Ki-67, alcian blue analysis of mucus from *IL-22* ^+/+^ and *IL-22* ^-/-^ mice (Scale bars, 200 μm). **(I)** Tunel assay analysis of colon tissue from *IL-22* ^-/-^ mice (Scale bars, 200 μm). **(J)** Western Blot analysis of pSTAT3, STAT3, PCNA, cleaved Caspase-3, and Caspase-3 from colonic epithelial cells in *IL-22* ^-/-^ mice. **(K)** Theβdiversity analysis of microbiota in *Stat3* ^△IEC^ mice between groups. **(L)** LEfSe analysis of differential bacteria in feces between the DSS group and ZY-312 group with different levels meeting a significant LDA threshold value of >3.5 in IL-22^-/-^ mice. NF.22KO indicates Control/IL-22^-/-^ group. DF.22KO indicates DSS/IL-22^-/-^ group. ZF.22KO indicates DSS+ZY-312/IL-22^-/-^ group. Data are presented as Mean ± SEM **(A, B, D, F, G)**. The P value was calculated using two-way ANOVA **(A, B)**. Statistical analysis was performed using one-way ANOVA **(D, F, G)**. *p < 0.05,**p < 0.01, ***p < 0.005, ****p < 0.001. ND indicates undetectable. NS indicates no significance.

Then we administered *B. fragilis* orally in a DSS-induced colitis *IL-22^-^* ^/-^ mice model. (**Figure S5**). We found that *IL-22^-^* ^/-^ mice receiving *B. fragilis* were more sensitive to DSS-induced colitis than wild-type (*IL-22^+^* ^/+^) mice receiving *B. fragilis*, evidenced by more weight loss (**Figure 5A**), a higher DAI score (**Figure 5B**), a shorter colon length (**Figures 5C, D**) and a higher HAI (**Figures 5E, F**; **Figure S6**). The RNA level of PCNA and Caspase-3 were comparable between the DSS group and the DSS+ZY-312 group in *IL-22^-^* ^/-^ mice (**Figure 5G**). Next, we verified whether *B. fragilis-*mediated pSTAT3 upregulation was impaired in IL-22^-/-^ mice. The immunohistochemical, immunofluorescence, and WB results showed that *B. fragilis* decreased pSTAT3 expression in *IL-22^-^* ^/-^ mice compared to *IL-22^+^* ^/+^ mice (**Figures 5H, J**). And *B. fragilis* did not increase pSTAT3 expression in colonic organoids with IL-22 neutralizing antibodies (**Figure S7C**). Synchronously, *B. fragilis* did not increase Ki-67 and PCNA levels, and mucus density in *IL-22^-^* ^/-^ mice compared to *IL-22^+^* ^/+^ mice (**Figures 5H, J**). CCK8 results showed that *B. fragilis* did not increase the survival rate of MC38 once blocking IL-22 (**Figure S7B**). Besides, *B. fragilis* did not decrease the cleaved Caspase-3 level and TUNEL-positive cell density compared to the DSS group in *IL-22^-^* ^/-^ mice (**Figures 5I, J**) and the colonic organoids with IL-22 neutralizing antibodies (**Figure S7C**). Besides, The β diversity and LEfSe analysis showed a similar microbiota composition between the DSS+ZY-312 group and the DSS group in *IL-22^-^* ^/-^ mice (**Figures 5K, L**). Together, these results demonstrated that *B. fragilis* promoted IL-22-mediated pSTAT3 phosphorylation to promote colonic mucosal proliferation and mucus secretion, and modulate intestinal microbiota in DSS-induced colitis.

### 3.4 *B. fragilis*-induced IL-22 secretion was mainly derived from ILC3

**Figure 6:**
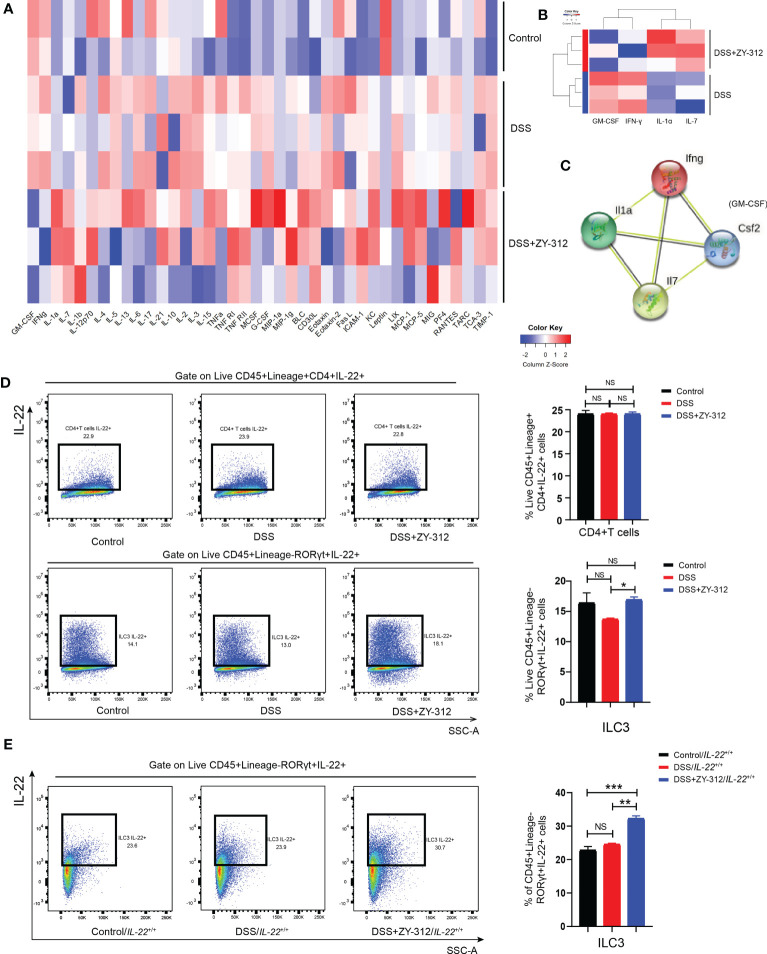
*B. fragilis* possibly promoted CLP-derived ILC3 to secrete IL-22 in DSS-induced colitis. **(A)** The heatmap analysis of inflammatory factors from colon tissue through protein microarray detection. Control group (N=2), DSS group (N=3), DSS+ZY-312 group (N=3). N, number of mice. Red represents up-regulation, and blue represents down-regulation. **(B)** The level of inflammatory factors including IL-7, IL-1α, GM-CSF, and IFN-γ showed significant differences between the DSS+ZY-312 group (N=3) and the DSS group (N=3). IL-7 (p value=0.0078), IL-1α (p value=0.0038), GM-CSF (p value=0.0173), IFN-γ (p value=0.0386), Red represents up-regulation, blue represents down-regulation. **(C)** The PPI (protein-protein interaction) analysis of DEGs (differentially expressed genes) between the DSS group and DSS+ZY-312 group. Network nodes represent proteins, edges represent protein-protein associations, colored nodes: query proteins and the first shell of interactors, white nodes: the second shell of interactors, and empty nodes: proteins of unknown 3D structure, contrary to a known 3D structure. **(D)** IL-22 secreting ILC3 (labeled with FVS-CD45+Lineage-RORγt+IL-22+) and CD4+T cells (labeled with FVS-CD45+Lineage+CD4+IL-22+) from CLP in C57BL/6 mice were analyzed by cell flow cytometry, and statistical analysis of cell percentage was showed (right panel). Control group (N=3), DSS group (N=13), DSS+ZY-312 group (N=11). N, number of mice. **(E)** Cell flow cytometry analysis of IL-22 secreting ILC3 in *IL-22* ^+/+^ mice. Control group/*IL-22* ^+/+^ (N=3), DSS group/*IL-22* ^+/+^ (N=3), DSS+*B. fragilis* group/*IL-22* ^+/+^ (N=3). N, number of mice. Data are presented as Mean ± SEM. Statistical analysis was performed using one-way ANOVA **(D, E)**. *p < 0.05,**p < 0.01, ***p < 0.005, NS indicates no significance.

IL-22 is mainly produced by ILC3 (35), CD4+ T cells, and Th17 cells (13). Next, we attempted to identify *B. fragilis*-promoted IL-22 secretion mainly derived from which type of immune cells in CLP. Initially, the protein microarray analysis demonstrated that *B. fragilis* promoted IL-7 and IL-1α expression, but did not affect the IL-1β, IL-6, IL-10, IL-17A, IL-23, TNF-α, or IFN-γ levels (**Figures 6A–C**). IL-7 was mainly derived from intestinal epithelial cells and promoted ILC3 development (36), and IL-1α was secreted from ILC3 for inflammation reaction (37, 38). It suggested that *B. fragilis* may promote ILC3, but not IL-17A secreting Th17 cells, to secrete IL-22 through IL-7 signaling. Using the flow cytometry gating strategy (**Figure S8**), we detected the cell percentage of IL-22-secreting ILC3 and CD4+ T cells. The cell flow cytometry results showed that *B. fragilis* prompted IL-22 production from ILC3 but not CD4+ T cells (**Figures 6D, E**). Next, we aimed to clarify whether the vitality of IL-22-secreting ILC3 was impaired under *B. fragilis* administration in *Stat3* ^△IEC^ mice. We found that *B. fragilis* promoted ILC3 to secrete IL-22 in *Stat3* ^fl/fl^ and *Stat3* ^△IEC^ mice, but not CD4+ T cells in *Stat3* ^△IEC^ mice (**Figure S9**). It suggests that *B. fragilis* promotes ILC3 cells to secrete IL-22 in DSS-induced colitis.

**Figure 7:**
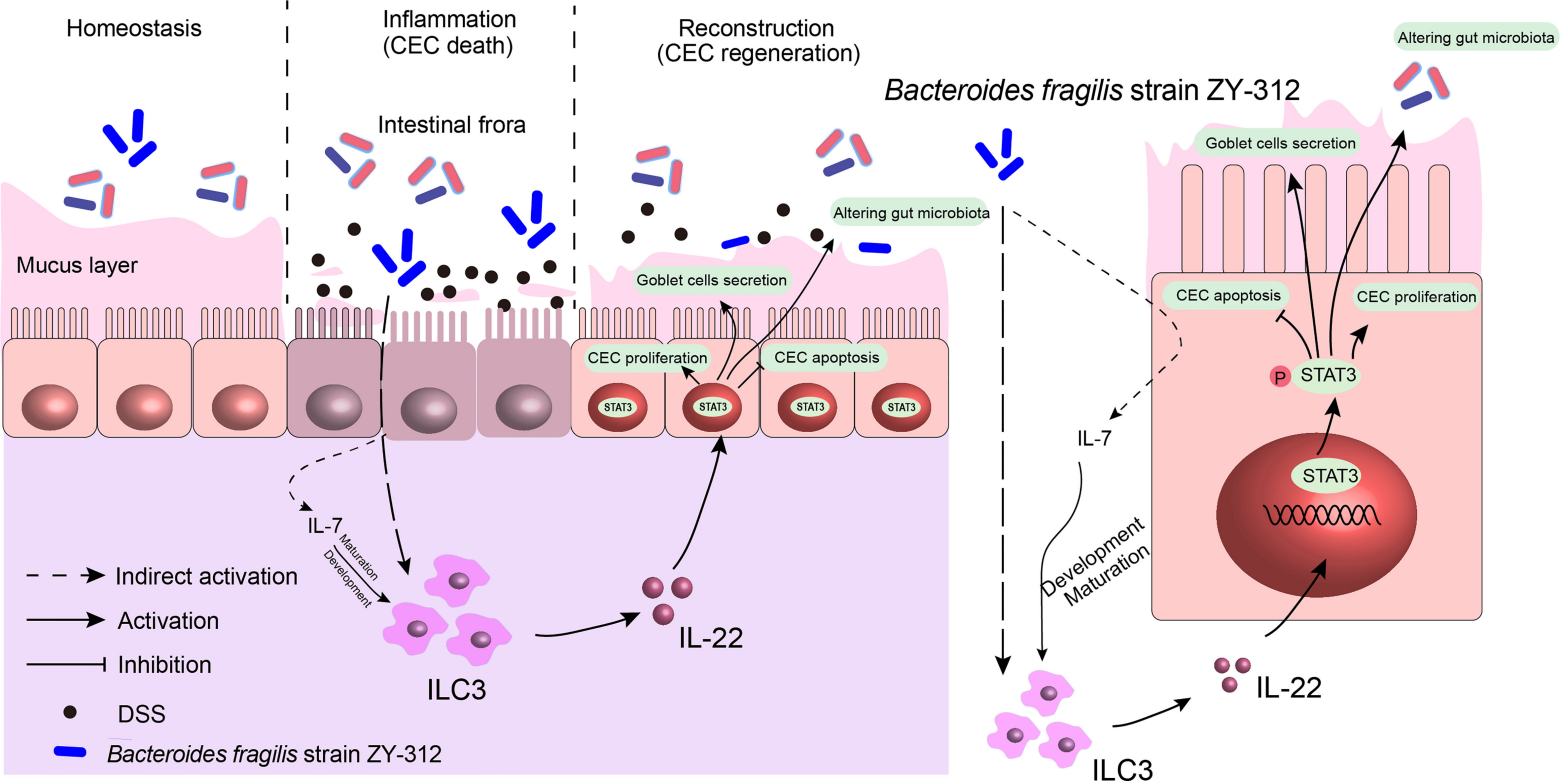
*B. fragilis* facilitates colonic mucosa regeneration in colitis *via* motivating the STAT3 pathway induced by IL-22 from ILC3 secretion. *B. fragilis* promoted CLP-derived ILC3 to secrete IL-22, then motivated the STAT3 signaling pathway, hence promoting colonic mucosa proliferation and mucus secretion, inhibiting apoptosis, and altering gut microbiota in DSS-induced colitis.

Overall, *B. fragilis* may indirectly motivate ILC3 (Meanwhile *B. fragilis* promote the maturation and development of ILC3 through IL-7 signaling) to secrete IL-22, followed by IL-22-induced STAT3 phosphorylation in the colonic mucosa, hence promoting colonic mucosa regeneration including cell proliferation and mucus secretion, and modulating intestinal microbiota in a DSS-induced colitis mouse model (**Figure 7**).

## 4 Discussion

Intestinal epithelial barrier impairment contributes to the amplification of the IBD-associated immuno-inflammatory response (39) and the imbalance of intestinal microbiota (40). Hence, it is a good candidate for IBD therapy by targeting epithelium barrier repair. Recently, the therapeutic effects of probiotics for promoting intestinal epithelial barrier restoration, inhibiting immunity-mediated inflammation reaction, and modulating intestinal microbiota are attracting attention in IBD applications. VSL#3 probiotic mixture induces remission in patients with active Ulcerative Colitis (25). *Lactobacillus reuteri* increases mucus density to exert a protective effect against DSS-induced colitis (41). *Lactobacillus rhamnosus* GG promotes intestinal epithelial cell proliferation and increases the diversity of gut microbiota in mice (42). And *Lactobacillus salivarius* Ls33-purified peptidoglycan induces the dendritic cell to secrete IL-10 through a NOD2-dependent manner to relieve colitis (43). In this study, we demonstrated that *B. fragilis* has a protective role in UC-like colitis by facilitating colonic mucosal regeneration including cell proliferation, mucus secretion, and altering the gut microbiota. It suggests the probiotic properties of *B. fragilis* in IBD which is consistent with the studies above.

It has been reported that some proliferation pathways, including STAT3 (28), p38 (29), and ERK (30), participate in intestinal mucosa proliferation and apoptosis in IBD. STAT3 is widely expressed in organs and tissues, and the phosphorylation of STAT3 motivates downstream genes related to proliferation and apoptosis, thus performing proliferation effects. A previous study has revealed that the motivation of STAT3 signaling promotes colonic mucus secretion in DSS-induced colitis in mice (28). Correspondingly, a probiotic *Lactobacillus* motivates STAT3 signaling to promote stem cell proliferation in DSS-induced colitis (44). High mobility group box-1 (HMGB1), a damage-associated molecular pattern protein related to microbe recognition, exerts an anti-inflammatory effect by increasing pSTAT3 expression in the colonic mucosa (45). In this study, we consistently found that *B. fragilis* motivated STAT3 phosphorylation to promote colonic mucosal regeneration including cell proliferation and mucus secretion. Moreover, considering that STAT3 is activated in acute colitis, and its expression gradually disappears during the chronic stage of the disease (46), here we speculate that *B. fragilis* motivates STAT3 signaling to facilitate colonic mucosa regeneration at the acute phase of DSS-induced colitis. Notably, it has been reported that the motivation of STAT3 signaling is linked to modulating gut microbiota in the diet-induced obesity model (47). Also, the activation of STAT3 signaling, induced by short-chain fatty acids, promotes antimicrobial peptide expression in intestinal epithelial cells, thereby affecting gut microbiota (48). Commensal bacteria protect against pathogen invasion by increasing mucus secretion and occupying the binding sites on the mucins to impede pathogen adhesion (49). Mucus has a scour and repelling effect on pathogenic bacteria, thus modulating gut microbiota (50). Based on that commensal *B. fragilis* motivates STAT3 signaling to promote mucus secretion, it indicates that *B. fragilis* may modulate gut microbiota through STAT3 signaling-mediated mucus secretion. In our study, we reveal that *B. fragilis* elevates the phylum level of *Firmicutes* and increases the abundance of butyric-producing *Allobaculum* in wild-type mice, while the abundance difference disappears once STAT3 is defective in intestinal epithelial cells.

Additionally, Upstream factors of STAT3 primarily include IL-22, IL-10, and IL-6 (33). IL-22 belongs to the IL-10 cytokine family and exerts anti-inflammatory effects (34). In this study, *B. fragilis* mainly promoted IL-22 production but has no apparent influence on IL-10, and IL-6. IL-22 promotes stem cell regeneration and mucus secretion in the intestinal epithelium (51, 52). Besides, IL-22 induces intestinal metabolites-related colonic mucosa saccharification to promote symbiotic bacteria colonization (15). And IL-22 mediates early host defense against attaching and effacing bacterial pathogens through antimicrobial peptide secretion (53). These roles suggest that IL-22 might mediate colonic mucosa regeneration and intestinal microbiota. A previous study has revealed a link between IL-22 and STAT3 signaling in intestinal epithelial renewal in DSS-induced colitis (44). In this study, we confirm that *B. fragilis* up-regulates IL-22/pSTAT3 axis to promote colonic mucosa regeneration and alter gut microbiota manifesting that *B. fragilis* promotes mucus secretion, meanwhile elevating α, and β diversity, and increases the abundance of butyrate-producing *unidentified clostridia* in wild-type mice, while the abundance difference disappears after IL-22 defects.

Referring to the cell source of IL-22, IL-22 is mainly derived from ILC3 (12), CD4+ T cells, and Th17 cells (13). ILC3 are innate immune cells and critical for IL-22 production (35). A previous study reported that ILC3 protects the host from enteric *Citrobacter rodentium* infection (54). Chun et al. reported a lower proportion of ILC3 in patients with IBD (55), suggesting that ILC3 may have a protective role in IBD. It has been reported that IL-7, expressed in intestinal epithelial cells (56), is required for ILC3 development and maintenance, and consequently for intestinal barrier defense (57). In this study, we confirm that *B. fragilis* may promote ILC3 to secrete IL-22 in a manner of IL-7 signaling. However, the signal transmission between *B. fragilis* and ILC3 remains unclear due to that ILC3 in mice does not have toll-like receptors to recognize intestinal microbiota (56). Potentially, intestinal microbiota-associated metabolites could activate aryl hydrocarbon receptors in ILC to secrete IL-22 (58, 59) It suggests that *B. fragilis-relative* metabolites may convey the signal to activate ILC3 to mediate IL-22 secretion. Therefore, we will investigate the mechanistic interactions between *B. fragilis* and ILC3 in our further studies.

## 5 Conclusion

In conclusion, we identify that ILC3-derived IL-22 is necessary for *B. fragilis* to activate the STAT3 signaling pathway, thereby facilitating colonic mucosal regeneration including cell proliferation and mucus secretion, and modulating intestinal microbiota. We expect that *B. fragilis* exerts beneficial effects in IBD *via* innate immunity-mediated colonic mucosa regeneration.

## Data availability statement

The datasets presented in this study can be found in online repositories. The names of the repository/repositories and accession number(s) can be found in **Supplementary Data Sheet 1**.

## Ethics statement

The animal experiments were approved by the ethics committee of the laboratory animal center of Southern Medical University (Ethics Committee Approval No. L2018053)

## Author contributions

WZ guided the experiment, and offered financial assistance. QZ was responsible for bacteria strain culture, animal experiment, colonic organoids construction, data analysis, and manuscript writing. HL was in charge of flow cytometry. JhX performed co-culture model *in vitro* assay. RH and BS were in charge of Molecular detection. YG revised the manuscript, and offered financial assistance. XA, JX, and XZ provided procedural advice, and revised the manuscript. YL and YW provided the *B. fragilis* strain ZY-312, revised the manuscript, and offered financial assistance. FZ designed the project and acquired funding. All authors contributed to the article and approved the submitted version.
