# Supplementary figures and images for: Bacteroides fragilis strain ZY-312 facilitates colonic mucosa regeneration in colitis via motivating STAT3 signaling pathway induced by IL-22 from ILC3 secretion
Source: Front Immunol. 2023 Apr 11;14:1156762. doi: 10.3389/fimmu.2023.1156762 (PMC10126674; doi:10.3389/fimmu.2023.1156762)

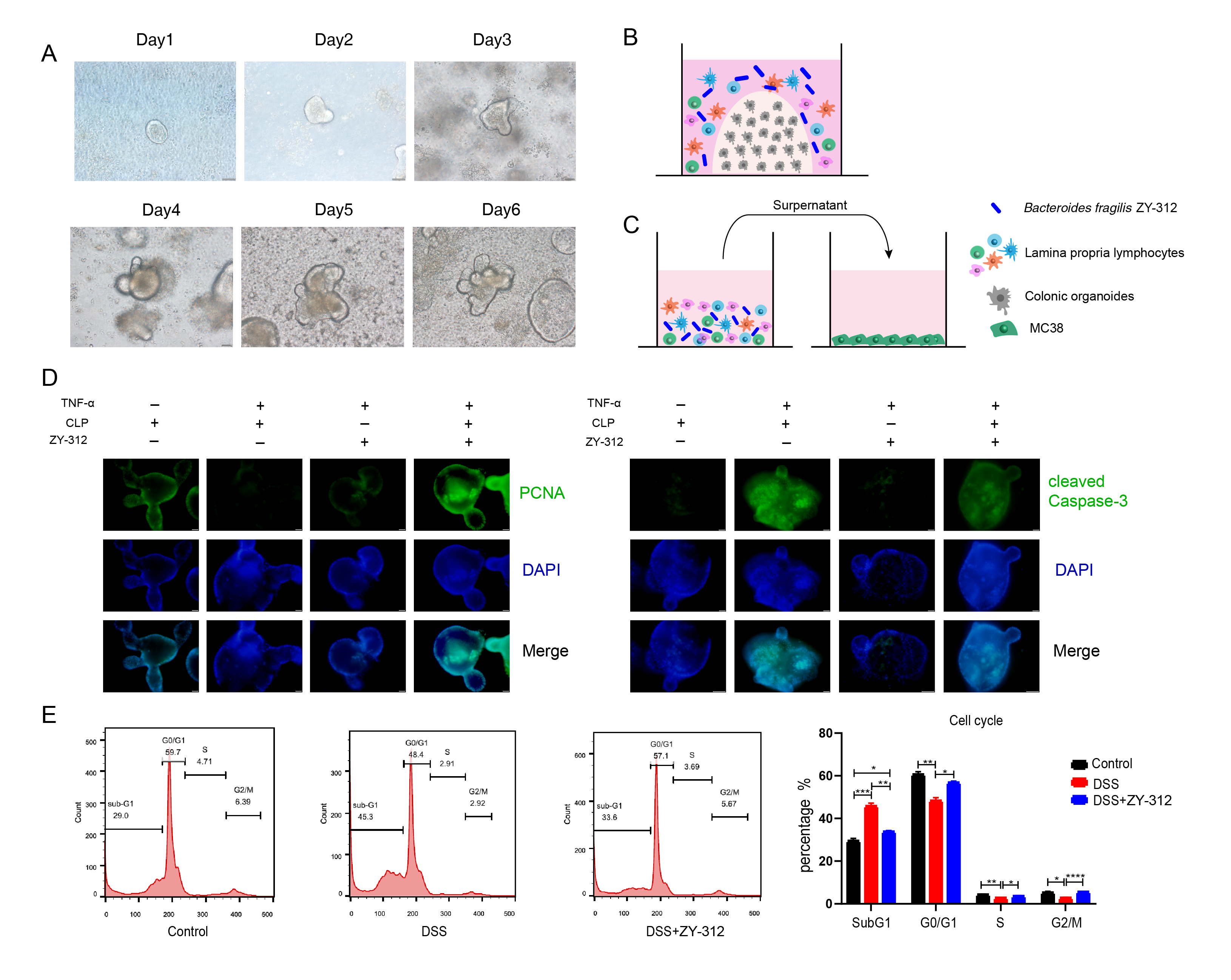

Supplement: Supplementary file 1 [file Image_1.tif]

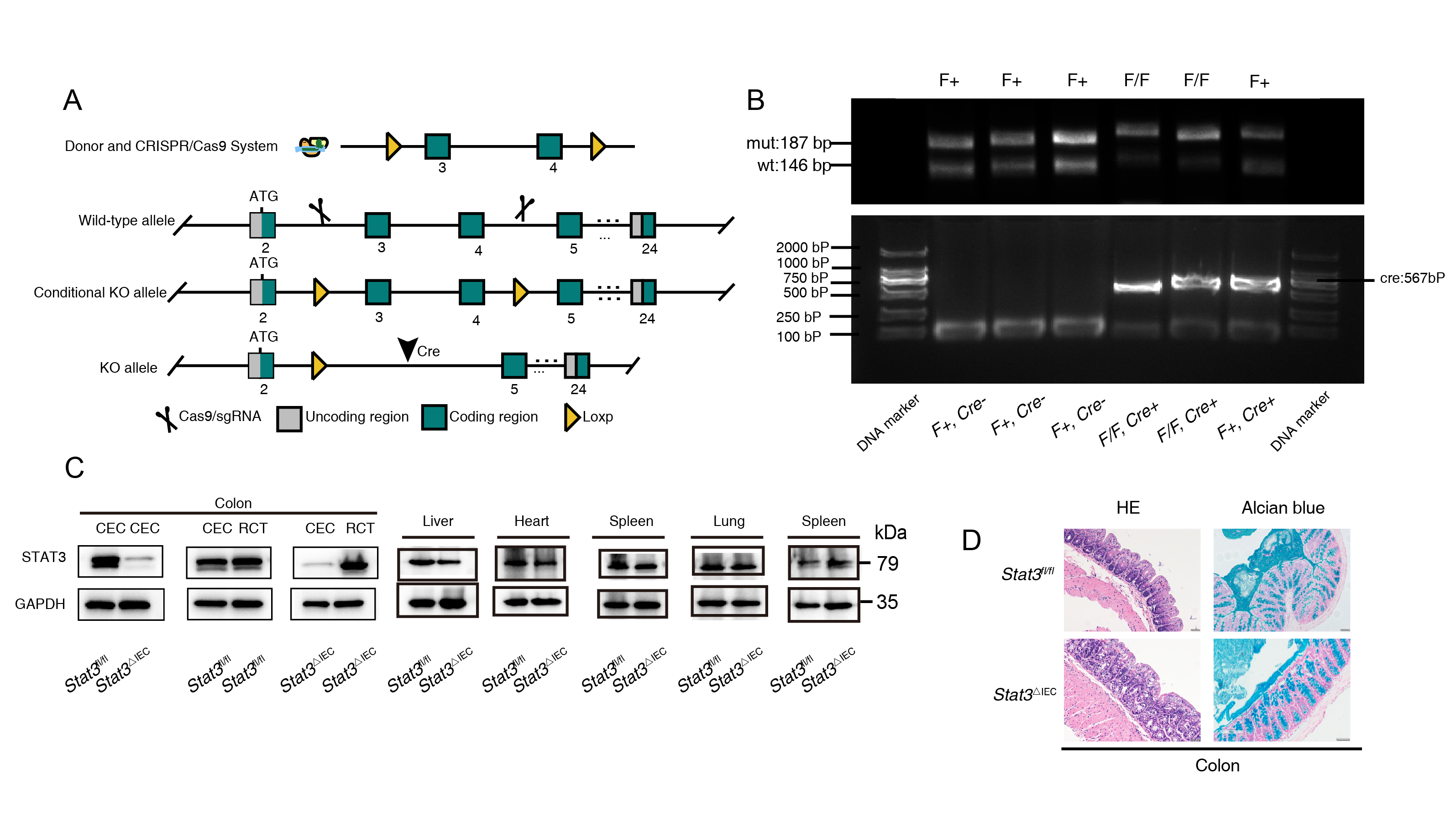

Supplement: Supplementary file 2 [file Image_2.tif]

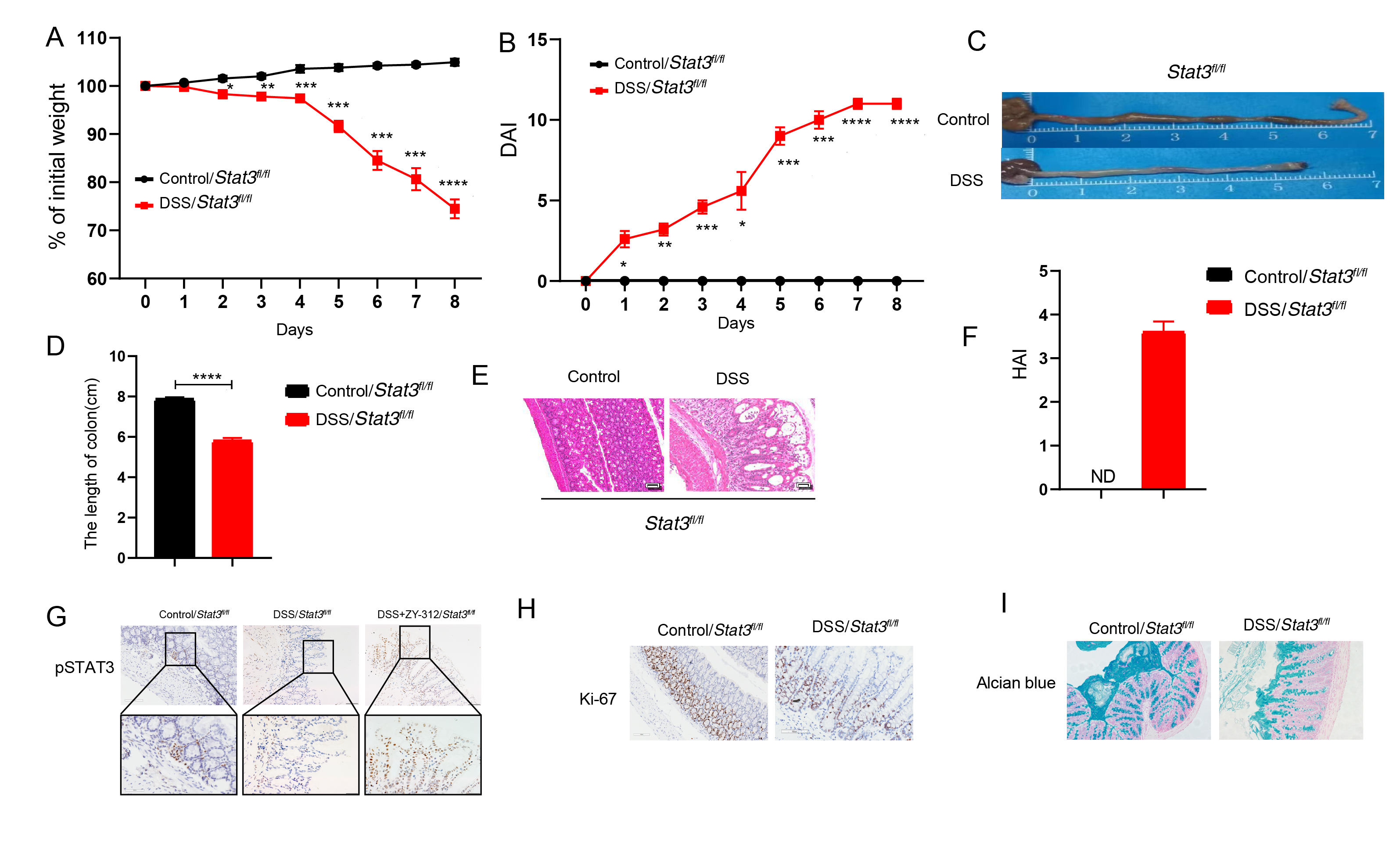

Supplement: Supplementary file 3 [file Image_3.tif]

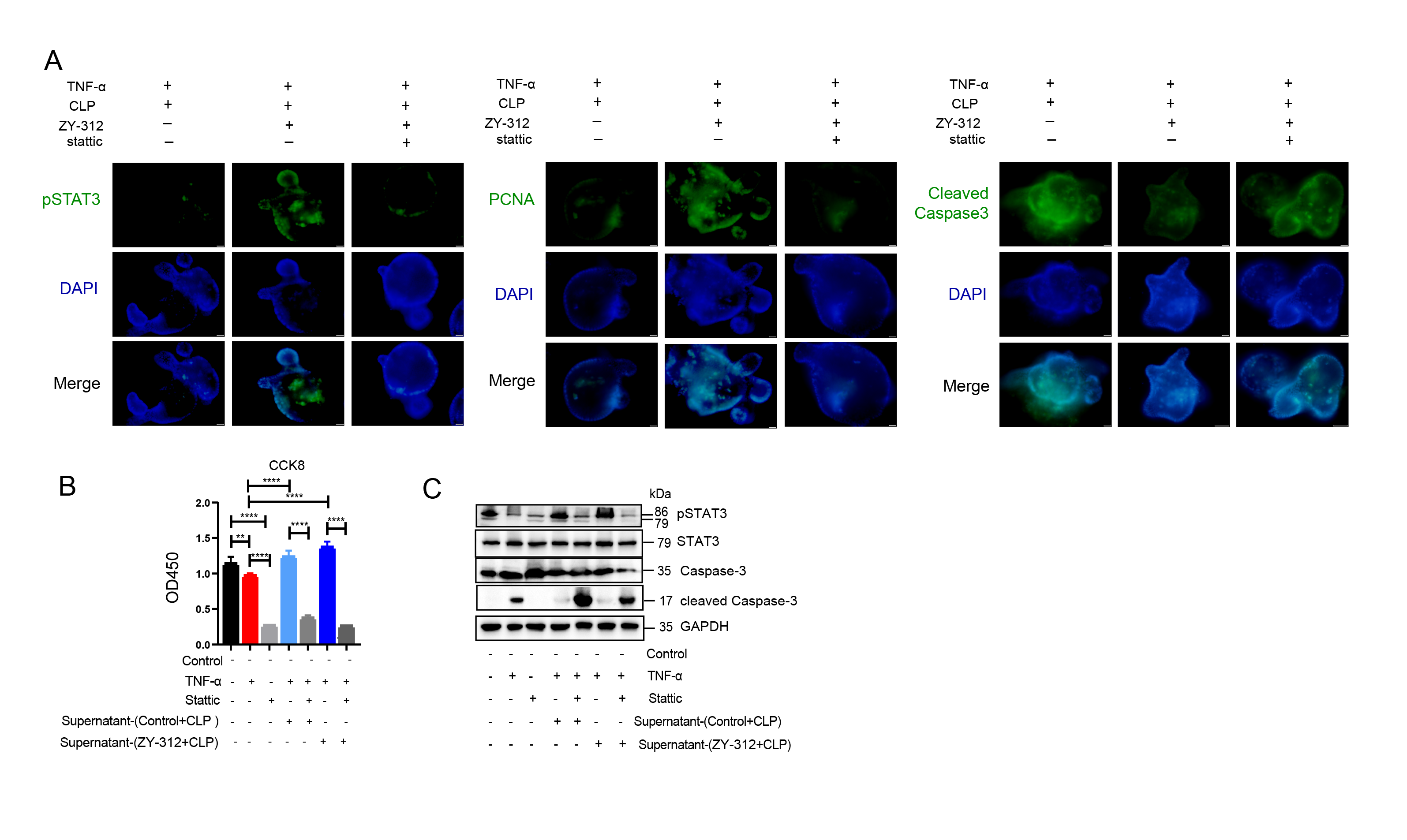

Supplement: Supplementary file 4 [file Image_4.tif]

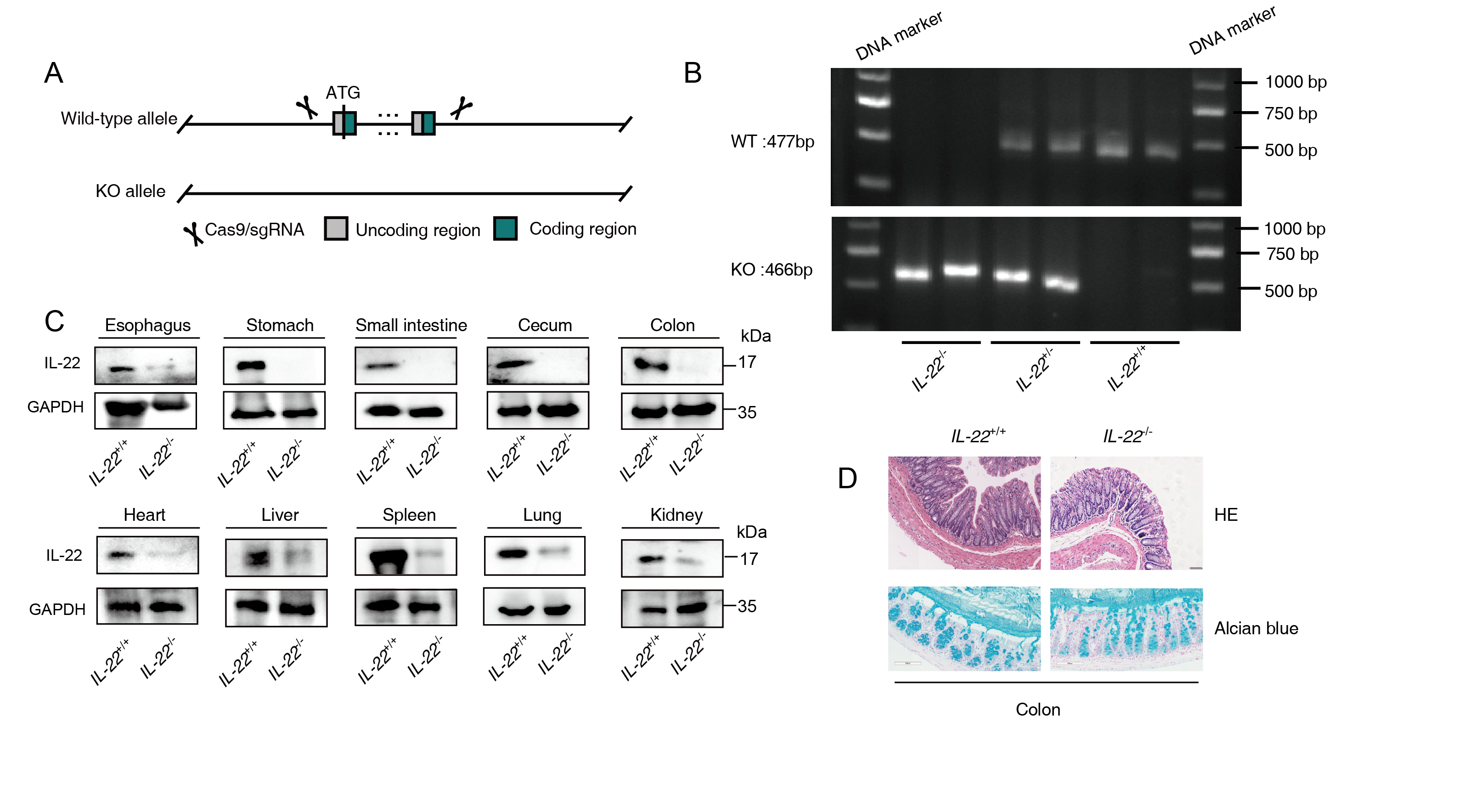

Supplement: Supplementary file 5 [file Image_5.tif]

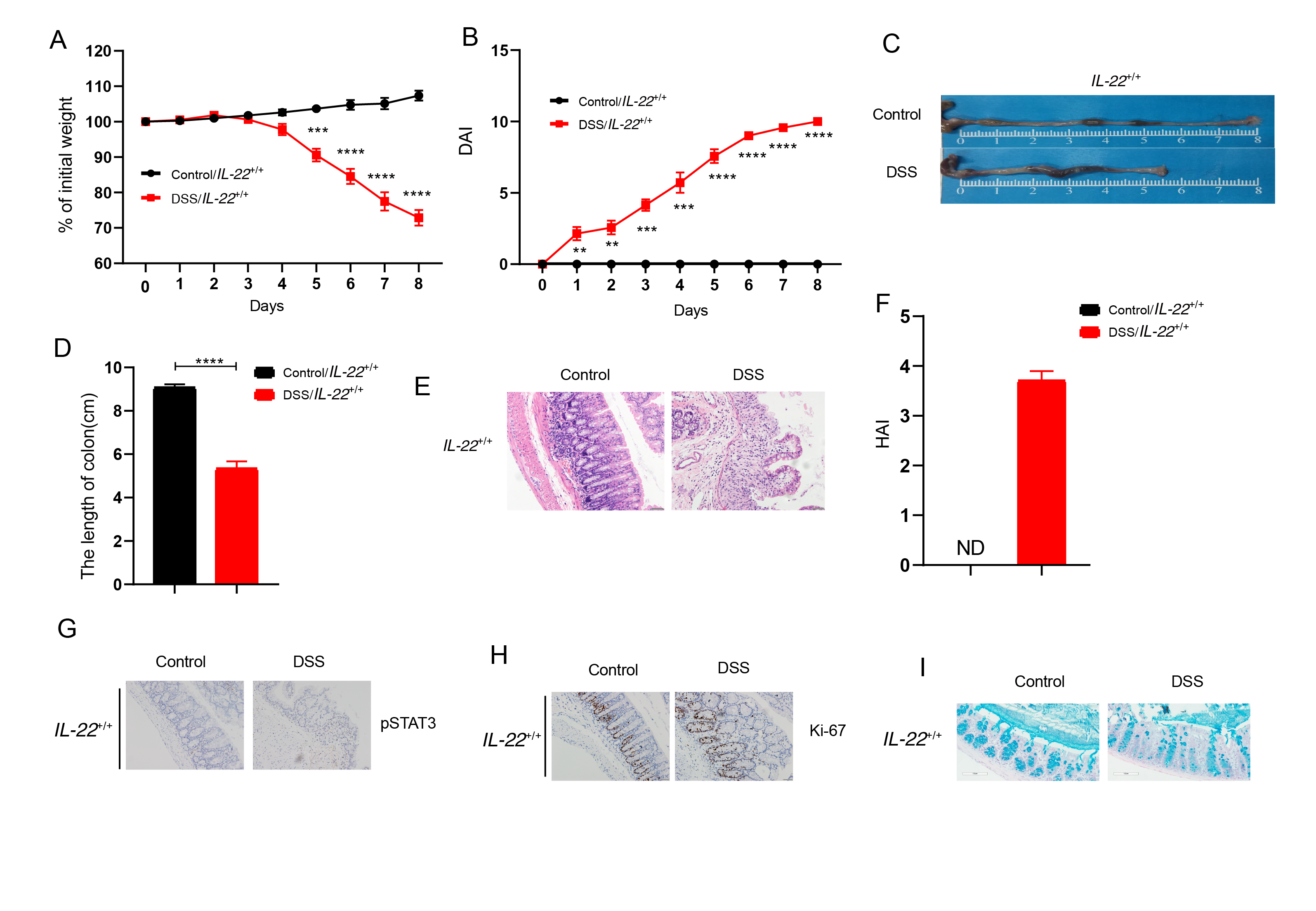

Supplement: Supplementary file 6 [file Image_6.tif]

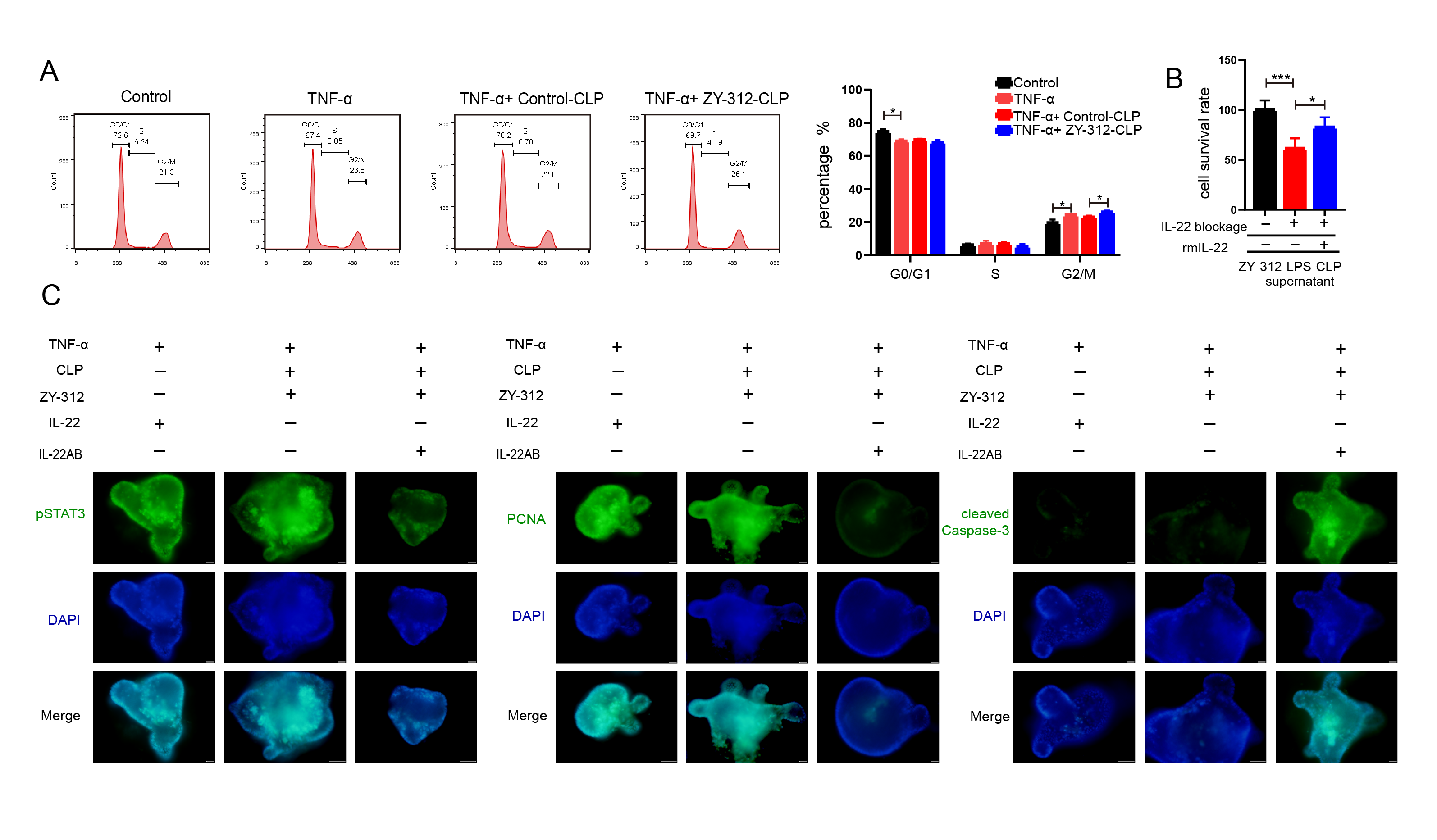

Supplement: Supplementary file 7 [file Image_7.tif]

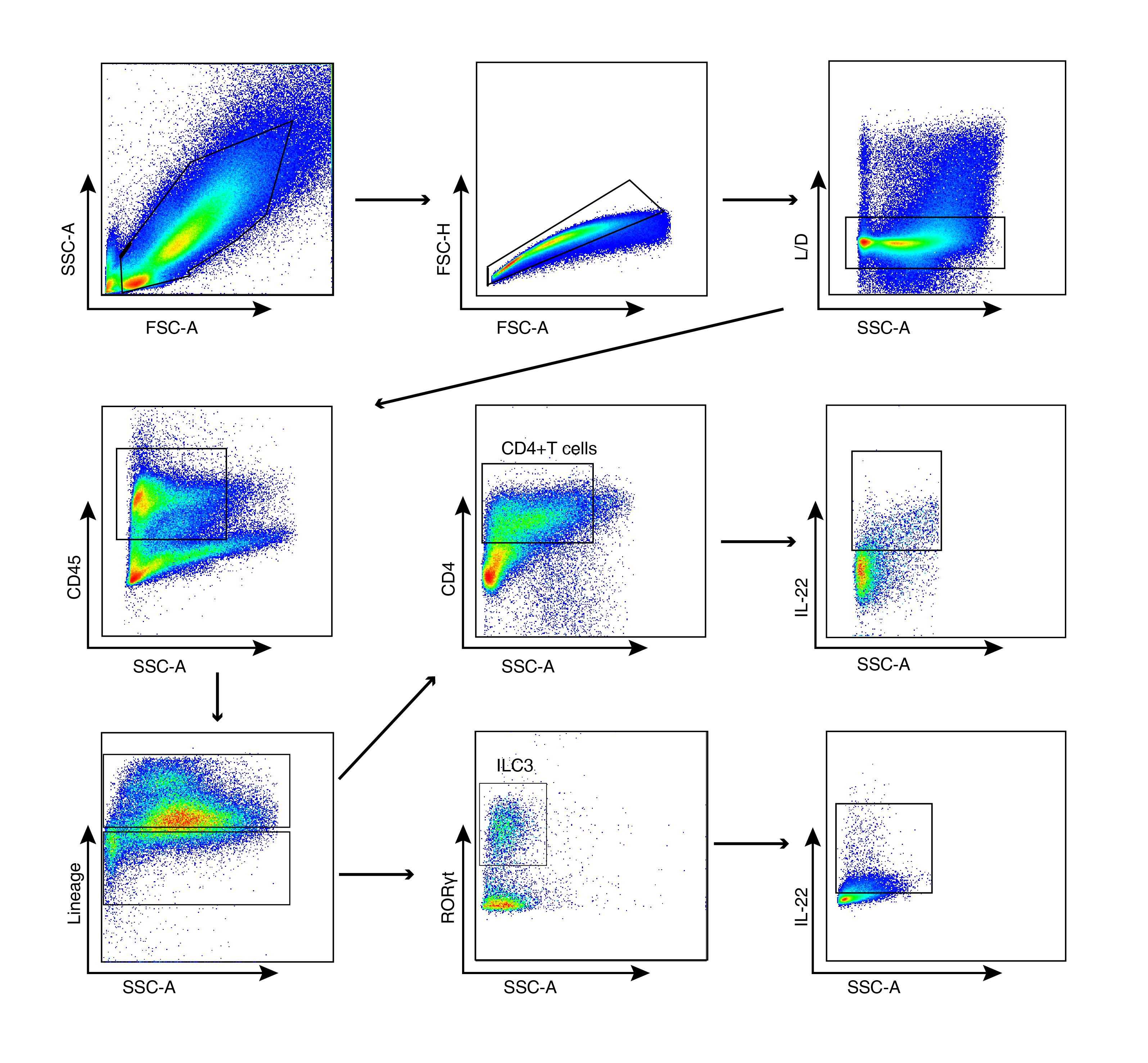

Supplement: Supplementary file 8 [file Image_8.tif]

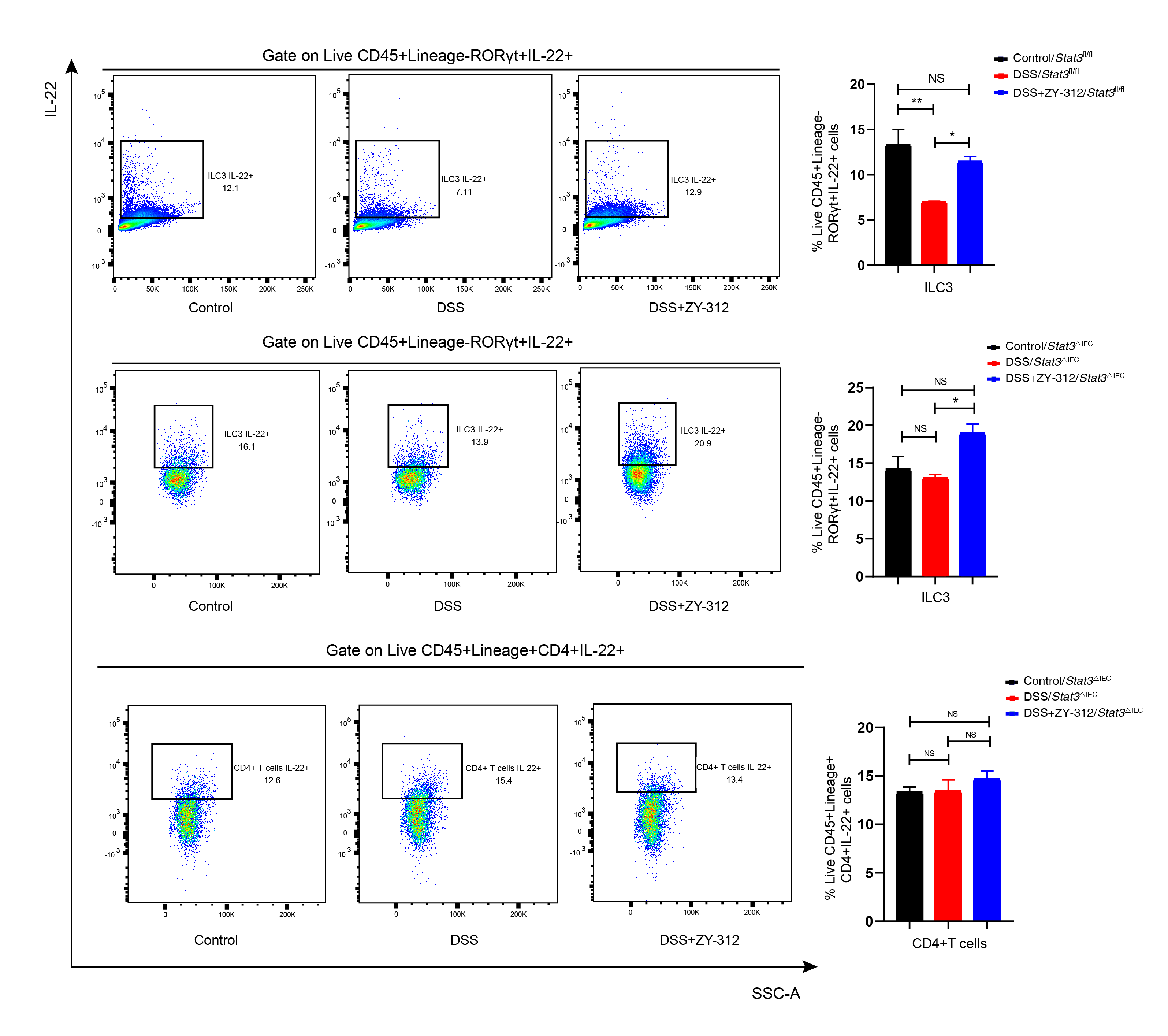

Supplement: Supplementary file 9 [file Image_9.tif]
