# Supplementary material for: Bacteroides fragilis strain ZY-312 facilitates colonic mucosa regeneration in colitis via motivating STAT3 signaling pathway induced by IL-22 from ILC3 secretion
Source: Front Immunol. 2023 Apr 11;14:1156762. doi: 10.3389/fimmu.2023.1156762 (PMC10126674; doi:10.3389/fimmu.2023.1156762)
Supplement: Supplementary file 10 [file DataSheet_1.docx]

**Supplemental information**

***Bacteroides fragilis* strain ZY-312 facilitates colonic mucosa regeneration in colitis via motivating STAT3 signaling pathway induced by IL-22 from ILC3 secretion.**

Wendi Zhang, Qian Zhou, Hongbin Liu, Jiahui Xu, Ruo Huang, Binhai Shen,Yandong

Guo, Xiuyun Ai, Jun Xu, Xinmei Zhao, Yangyang Liu, Ye Wang, Fachao Zhi.

All the raw data has been uploaded to the Jianguoyun repository recommended by the magazine. The following is the link to the raw data, <https://www.jianguoyun.com/p/DVzEBtAQpt70ChiM7NYEIAA.>

The 16srRNA sequencing data were submitted to NCBI(SRA (Sequence Read Archive ) <http://www.ncbi.nlm.nih.gov/sra/),> and the number is SUB12696043.

**Inventory of Supplemental Material:**

# Supplementary Figures 1-7

## Fig.S1. The development process of colonic organoids from day1 to day7, and the description of the co-culture models. (A) Colonic crypts developed into mature organoids on day 6 (Scale bars, 50 μm). (B) The construction of the co-culture model. The co-culture model, including colonic crypts (150/well) and CLP (10^7^ cells/well), was added with *B. fragilis* (10^4^ CFU/well) after TNF-α (60ng/ml) induced inflammation environment for 24h. (C) The description of the MC38 co-culture model. MC38 was stimulated with the supernatant after *B. fragilis* co-culturing with CLP. (D) Immunofluorescence analysis of PCNA and cleaved Caspase-3 in colonic organoids from co-culture model (Scale bars:50 μm). The co-culture model was added with (+) or without (-) *B. fragilis* strain ZY-312 (10^4^ CFU/well) after TNF-α (60ng/ml) induced inflammation for 24 hours in vitro. (E) Cell cycle flow cytometry analysis of colonic epithelial cells. Data are presented as Mean ± SEM. Statistical analysis was performed using two-way ANOVA. *p < 0.05,**p < 0.01, ***p < 0.005, ****p < 0.001.

## Fig.S2. Generation and evaluation of floxed alleles of STAT3 in colonic mucosa in mice. (A) CRISPER Cas9 strategy of STAT3 conditional defect in intestinal mucosa in mice. (B) Polymerase chain reaction (PCR) was performed on DNA prepared from tail biopsies, using a mixture of primers to amplify the following two bands: 187 bp (floxed allele, lanes 1,2,3,4,5, and 6), 146 bp (Wild type allele, lanes 1,2,3, and 6), 567 bp (cre allele, lanes 4,5 and 6) and analysis of cre and flox from STAT3 conditional gene knockout mice. (C) Western Blot analysis of the level of STAT3 from colonic epithelial cells (CEC) and rest colon layer tissue (RCT), and organs in mice. (D) Histopathology analysis of colon, and alcian blue analysis of mucus from STAT3 conditional gene knockout mice (*Stat3*^△IEC^ mice) (Scale bars, 200 μm).

## Fig.S3. *B. fragilis* alleviated DSS-induced colitis in *Stat3*^fl/fl^ mice. (A-B) Percent of weight loss (A) and DAI (B) was monitored daily starting from DSS administration and presented relative to the initial body weight in *Stat3*^fl/f^ (wild-type, WT) mice. Control group (N=6), DSS group (N=5). (C) Representative of the morphology of the colon. (D) The significant analysis of colon length. (E-F) Histopathological morphology (E) and HAI (F) of the colon (Scale bars, 200 μm). (G) Immunochemistry analysis of the level of pSTAT3 (scale bars: upper 200 μm, below 50 μm). (H) Immunochemistry analysis of the level of Ki-67 (scale bars, 200 μm). (I) Alcian blue analysis of mucus. (scale bars: upper 200 μm). N, the number of mice. Data are presented as Mean ± SEM. The P value was calculated using a Student’s t-test (A, B, D, F). *p < 0.05,**p < 0.01, ***p < 0.005, ****p < 0.001.

## Fig.S4. *B. fragilis* motivated STAT3 phosphorylation to promote proliferation and inhibit apoptosis in vitro. (A) Immunofluorescent analysis of pSTAT3 , PCNA, and cleaved Caspase-3 in colonic organoides from the co-culture model. (Scale bars, 200 μm). (B) CCK8 assay analysis of MC38 proliferation level. MC38 co-culture model was added with STAT3 inhibitor (stattic, 20 μM) after TNF-α (20ng/ml) stimulation for 12h. Data are presented as Mean ± SEM. Statistical analysis was performed using one-way ANOVA. *p < 0.05,**p < 0.01, ***p < 0.005, ****p < 0.001. (C) Western blot analysis of pSTAT3, STAT3, PCNA, Cleaved Caspase-3, and Caspase-3 in MC38.

## Fig.S.5 The identification of IL-22 system gene knockout mice. (A) CRISPER Cas9 strategy of IL-22 system deficient mice. (B) PCR was performed on DNA prepared from tail biopsies, using a mixture of primers to amplify the following two bands: 477 bp (Wild type allele, lanes 3,4,5 and 6), 466bp (knockout allele, lanes 1,2,3 and 4) from IL-22 gene knockout mice. (C) Western Blot analysis of IL-22 expression in organs. (D) Histopathology analysis of colon, and alcian blue analysis of mucus in wild-type mice (*IL-22*^+/+^ mice) and IL-22 gene knockout mice (*IL-22*^-/-^ mice) (Scale bars, 50 μm).

## Fig.S6. *B. fragilis* alleviated DSS-induced colitis in *IL-22*^+/+^ mice. (A-B) Percent of weight loss (A) and DAI (B) was monitored daily starting from DSS administration and presented relative to the initial body weight in *IL-22*^+/+^ (wild-type, WT) mice. Control group (N=6), DSS group (N=5). (C) Representative of the morphology of the colon. (D) The significant analysis of colon length. (E-F) Histopathological morphology (E) and HAI (F) of the colon (Scale bars, 200 μm). (G) Immunochemistry analysis of the level of pSTAT3 (scale bars: upper 200 μm, below 50 μm). (H) Immunochemistry analysis of the level of Ki-67 (scale bars, 200 μm). (I) Alcian blue analysis of mucus. [scale bars: upper 200 μm]. N, the number of mice. Data are presented as Mean ± SEM. The P value was calculated using a Student’s t-test (A, B, D, F). *p < 0.05,**p < 0.01, ***p < 0.005, ****p < 0.001.

## Fig.S7. *B. fragilis* up-regulated IL-22/pSTAT3 pathway to facilitate proliferation, and inhibit apoptosis in vitro. (A) The cell cycle (G0/G1 phase, S phase, G2/M phase) flow cytometry of MC38. (B) The CCK8 analysis of the survival rate of MC38, MC38 co-culture model was stimulated with IL-22 blocking. IL-22 recombinant protein (rmIL-22). (C) Immunofluorescence analysis of pSTAT3, PCNA, and Cleaved Caspase-3 in colonic organoids from co-culture model (Scale bars, 200 μm). The co-culture model was added with (+) or without (-) IL-22 (IL-22 recombinant protein, 5ng/ml), *B. fragilis* strain ZY-312 (10^4^ CFU/well), and IL-22AB (IL-22 neutralizing antibody, 100ng/ml) after TNF-α (60ng/ml) induced inflammation for 12h in vitro. Data are presented as Mean ± SEM. Statistical analysis was performed using one-way ANOVA (A,B). *p < 0.05,**p < 0.01, ***p < 0.005, ****p < 0.001.

## Fig.S8. Gating strategy for flow cytometry analysis of the CLP-derived CD4+T cells and ILC3 for IL-22 secretion.

## Fig.S9. IL-22 production from CLP in *Stat3*^△IEC^ mice. Cell flow cytometry analysis of IL-22 production from CLP-derived ILC3 (labeled with FVS-CD45+Lineage-RORγt+IL-22+) in *Stat3*^fl/fl^ and *Stat3*^△IEC^ mice. Control group (N=3), DSS group (N=6),DSS+ZY-312 group (N=8) from *Stat3*^fl/fl^ mice. Control group (N=7), DSS group (N=6), DSS+ZY-312 group (N=11) from *Stat3*^△IEC^ mice. Cell flow cytometry analysis of IL-22 production from CD4+T cells (labeled with FVS-CD45+Lineage+CD4+IL-22+) in *Stat3*^△IEC^ mice. Control group (N=3), DSS group (N=3), DSS+ZY-312 group (N=3). N, the number of mice. Data are presented as Mean ± SEM. Statistical analysis was performed using one-way ANOVA. *p < 0.05,**p < 0.01.

# Supplementary Tables 1-7

## Table S1. Detailed scoring method of Disease Activity Index.

| Score | Weight loss | Stool character | Occult or rectal bleeding |
| --- | --- | --- | --- |
| 0 | No change | Normal | Negative |
| 1 | 1-5% |  |  |
| 2 | 5-10% | Soft stool | Positive occult blood in stools |
| 3 | 10-20% |  |  |
| 4 | >20% | Diarrhea | Bloody stools |

## Table S2. Detailed scoring method of histopathology associated index.

| Scores | Colonic epithelial damage | Inflammatory cell infiltration | | |
| --- | --- | --- | --- | --- |
|  |  | mucous layer | submucous layer | seromuscular layer |
| 0 | Normal | Normal | Normal | Normal |
| 1 | Hyperplasia, irregular crypts, and goblet cells disappeared | mild | mild to moderate | moderate to severe |
| 2 | Medium and mild crypt disappeared (10%-50%) | moderate | severe | / |
| 3 | Severe crypts disappear (50%-90%) | severe | / | / |
| 4 | The crypt disappeared completely without ulcer formation | / | / | / |
| 5 | Small to medium ulceration (ulcer width< 10 crypt width) | / | / | / |
| 6 | Large ulcer (ulcer width ≥10 crypt width) | / | / | / |

## Table S3. The antibodies and reagents used in this study.

| REAGENTS or ANTIBODIES | SOURCE | IDENTIFIER |
| --- | --- | --- |
| Anti-pSTAT3 (Tyr705) | Cell Signaling Technology | CST#9145 |
| Anti-STAT3 (Tyr705) | Cell Signaling Technology | CST#4904 |
| Anti-IL-22 | Bioss | bs-2623R |
| Anti-ERK | Cell Signaling Technology | CST#4695 |
| Anti-pERK | Cell Signaling Technology | CST#4370 |
| Anti-P38 | Cell Signaling Technology | CST#9212 |
| Anti-pP38 | Cell Signaling Technology | CST#4511 |
| Anti-GAPDH | Cell Signaling Technology | CST#5174 |
| Anti-PCNA | Cell Signaling Technology | CST#13110S |
| Anti-Caspase3 | Novus | CPP32 4-1-18 |
| Anti-Cleaved-Caspase3 | Cell Signaling Technology | CST#9661S |
| KI-67 | abcam | Ab16667 |
| ServicebioTM Fluorescein (FITC) Tunel Cell Apoptosis Detection Kit | Servicebio | G1501-20 |
| PAS | Biossci | BP038 |
| Alcian blue | Biossci | BP040 |
| Anti-mouse IgG, HRP-linked Antibody | Beyotime | A0192 |
| Anti-rabbit IgG, HRP-linked Antibody | Beyotime | A0208 |
| Goat Anti-Rabbit IgG Antibody [H+L], FITC Conjugated | Beyotime | A0556 |
| DAPI | Beyotime | C1005 |
| IL-22 Mouse Uncoated ELISA Kit | Thermo Fisher Scientific | 88-7422-86 |
| IL-6 Mouse Uncoated ELISA Kit | Thermo Fisher Scientific | 88-7064-88 |
| TNF alpha Mouse Uncoated ELISA Kit | Thermo Fisher Scientific | 88-7324-88 |
| IL-17 Mouse Uncoated ELISA Kit | Thermo Fisher Scientific | 88-7324-88 |
| IL-10 Mouse Uncoated ELISA Kit | Thermo Fisher Scientific | 88-7064-88 |
| Mouse IFN gamma Uncoated ELISA Kit | Thermo Fisher Scientific | 88-7314-88 |
| Mouse IL-1β Uncoated ELISA Kit | Thermo Fisher Scientific | 88-7013-22 |
| IL-23 Mouse Uncoated ELISA Kit | Thermo Fisher Scientific | 88-7230-22 |
| DSS | MP Biomedicals | 9011-18-1 |
| Mouse IL-22 Affinity Purified Polyclonal Ab (25 ug) | R＆D system | AF582-SP |
| Murine IL-22 | Peprotech | 210-22-10 |
| TNF-α | Peprotech | 315-01A-20 |
| LPS | Beyotime | ST1470 |
| Mouse Inflammation Array Q1 | Raybiotech | QAM-INF-1-1 |
| Alexa Fluor® 700 Rat Anti-Mouse CD45 | BD Biosciences | 560510 |
| Fixable Viability Stain 780 | BD Biosciences | 565388 |
| Horizon™ BV605 Rat Anti-Mouse CD4 | BD Biosciences | 563151 |
| Pharmingen™ PerCP-Cy™5.5 Mouse Lineage Antibody Cocktail,with Isotype Control | BD Biosciences | 561317 |
| Horizon™ BV421 Mouse Anti-Mouse RORγt | BD Biosciences | 562894 |
| BD Pharmingen™ PE Mouse anti-Mouse IL-22 | eBioscience | 4317946 |
| Gentle Cell Dissociation Reagent | Stem cell | 07174 |
| IntestiCult OGM Mouse Kit | Stem cell | 06005 |
| Matrigel® Growth Factor Reduced (GFR) Basement Membrane Matrix, Phenol red-free, LDEV-free | Corning | 356231 |
| DMEM / F12 MEDIA with 15 mM HEPES, | Stem cell | 36254 |
| Lamina Propria Dissociation Kit | Miltenyi Biotec | 130-097-410 |
| Stattic | Selleck | S7024 |
| FITC-labeled Goat Anti-Rabbit IgG (H+L) | Beyotime | A0562 |

## Table S4. Serum microarray cytokines

| Microarray cytokines | | | | | | | |
| --- | --- | --- | --- | --- | --- | --- | --- |
| GM-CSF | IL-4 | IL-10 | TNFa | BLC | IL-12p70 | IL-21 | TIMP-1 |
| IFNg | IL-5 | IL-2 | TNF RI | CD30L | LIX | MCP-5 | Leptin |
| IL-1a | IL-13 | IL-3 | TNF RII | Eotaxin | MCP-1 | MIG | PF4 |
| IL-7 | IL-6 | IL-15 | MCSF | Eotaxin-2 | MIP-1a | ICAM-1 | RANTES |
| IL-1b | IL-17 | TCA-3 | G-CSF | Fas L | MIP-1g | KC | TARC |

## Table S5.Primers used in reverse transcriptase polymerase chain reaction

| Primer Name | Sequence（5’-3’） |
| --- | --- |
| Vil1-ProF1 | GTGTTTGGTTTGGTTTCCTCTGCATAAGA |
| Cre5R1 | GCAGGCAAATTTTGGTGTACGGTCA |
| 19436 | TTGACCTGTGCTCCTACAAAAA |
| 19437 | CCCTAGATTAGGCCAGCACA |
| JS01715-Il22-D5-5tF1 | GGTTCTTAAAGGAGTTCTGACCG |
| JS01715-Il22-D3-3tR1 | ACCTCTTGTCATCACAGGCTCC |
| JS11715-Il22-wt-tF2 | TATGAAACATATCCACGAGGAGC |
| JS11715-Il22-wt-tR2 | TTCACTGTCTCCTTCAGCCTTCTG |

## Table S6. Primers used in reverse transcriptase polymerase chain reaction

| Primers and related sequence | SEQUENCE |
| --- | --- |
| GAPDH (mouse) | Forward: GGAGAAACCTGCCAAGTATGA |
|  | Reverse: TCCTCAGTGTAGCCCAAGA |
| PCNA (mouse) | Forward: ATCGTGAATCGGGGGACCTT |
|  | Reverse: CGGAGTTGTGGCGACTAGAT |
| Caspase3 (mouse) | Forward: GAGCTTGGAACGGTACGCT |
|  | Reverse: TCCGTACCAGAGCGAGATGA |

# Supplementary Materials and Methods

## Gene knockout mouse model construction

STAT3 conditional gene knockout mice were first constructed by B6/STAT3^flox/flox^ mice, then mated with tool mice B6/ JNju-TG (Pvillin-Cre) D/Nju mice. mice with floxed Stat3 alleles were crossed to transgenic mice expressing the Cre-recombinase under the control of the Villin promoter. Particularly, according to the design principle of CRISPR/Cas9-CKO mice, a loxP element was placed on either side of the target region of the target gene (*Stat3*, the intestinal epithelial region of mice). After Cre expression, the target region anchored by the loxP element, i.e., the Flox region, would be deleted, resulting in subsequent exon code shifting mutations. The protein translation was terminated in advance to knock out the target gene Stat3 in mouse intestinal epithelium. The construction of *Stat3*^△IEC^ mice was divided into two stages. sgRNA and donor vectors were designed and constructed in vitro. Microinjection and transplantation of fertilized eggs, birth and identification of F0 generation mice, propagation of positive F0 generation mice, and birth and identification of F1 generation mice were performed in vivo. Secondly, we used the villin-cre from Jackson laboratory. The vil1-cre mice express cre recombinase in villus and crypt epithelial cells of the small and large intestines and may be useful in studies of intestinal organogenesis. In contrast to Villin-cre transgenic mice from founder line 1000 (Stock No. 021504) that are reported to be absent of Cre recombinase activity in gonads, these Villin-cre transgenic mice from founder line 997 are reported to have a very low level (<1%) of cells with cre recombinase activity in the testes. The specific method was the following: <https://www.jax.org/strain/004586.> The STAT3 conditional knockout mouse was genotyped with the set of primers Forward-WT:TTGACCTGTGCTCCTACAAAAA and Reverse-WT: CCCTAGATTAGGCCAGCACA(Table S5). These primers yield a 187 bp band for floxed allele and a 146 bp band for wild-type locus. The gene editing strategies and identification results of STAT3 conditional gene knockout mice were shown (Fig. S2A,2B). There was no overt phenotype after STAT3 ablation in colonic epithelial (Fig. S2).

IL-22 system gene knockout mice were constructed by CRISPR/Cas9 gene editing technology in C57/BL6. The brief process is as follows: sgRNA was transcribed in vitro. Cas9 and sgRNA were microinjected into the fertilized eggs of C57BL/6J mice. Fertilized eggs were transplanted to obtain positive F0 mice which were confirmed by PCR and sequencing. The IL-22 system gene knockoutmice were genotyped with the set of primers-Forward-WT: TATGAAACATATCCACGAGGAGC and Reverse-WT: TTCACTGTCTCCTTCAGCCTTCTG (Table S5). These primers yield a 466 bp band for wild-type locus. The gene editing strategies and genetic identification results of IL-22 system gene knockout mice were shown (Fig. S5A-B). There was no overt phenotype after IL-22 ablation in mice (Fig. S6).

## Cell Staining for Flow Cytometry

Live-dead labeling with FVS (BD Biosciences) and only live cells were analyzed. Fixed cells were permeabilized using a permeabilization buffer (eBioscience). For analysis of ILC3s and CD4+T cells, live colonic lamina propria immune cells were stained with anti-45,anti-CD4,anti-Lineage, and anti-RORγt as described (1). To detect IL-22 from ILC3s and CD4+T cells, colonic lamina propria immune cells were incubated with a 500x cell stimulation cocktail (plus protein transport inhibitors (eBioscience) in complete media at 37°C for 6 hours. Cells were stained with anti-CD45, anti-CD4, and anti-Lineage, Cells were further stained intracellularly with anti-RORγt and anti-IL-22. Results were read by a Flow Cytometer (Aria III, BD bioscience). Data were analyzed by Flowjo 10.0 software. Commercially available antibodies used in the flow cytometry experiments above are listed in Table S3. The Pharmingen™ PerCP-Cy™5.5 Mouse Lineage Antibody Cocktail has been designed to react with cells from the major hematopoietic lineages, such as T lymphocytes, B lymphocytes, monocytes/macrophages, NK cells, erythrocytes, and granulocytes. This pre-diluted Cocktail of five PerCP-Cy™5.5-conjugated antibodies is designed to label lineage marker-positive cells for exclusion to facilitate the flow cytometric identification of lineage marker-negative hematopoietic progenitors in mouse bone marrow. Components include clone 145-2C11, which recognizes Mouse CD3e; M1/70, which recognizes CD11b; RA3-6B2, which recognizes CD45R/B220; TER-119, which recognizes Ly-76, mouse erythroid cells; and RB6-8C5, which recognizes Ly-6G and Ly-6C. PerCP-Cy™5.5 Mouse Lineage Isotype Control Cocktail contains equivalent concentrations of isotype-matched negative-control immunoglobulin.

**Supplementary References**

1. Serafini N, Klein Wolterink RG, Satoh-Takayama N, Xu W, Vosshenrich CA, Hendriks RW, et al. Gata3 drives development of RORγt+ group 3 innate lymphoid cells. *J Exp Med* (2014) 211(2):199-208. doi:10.1084/jem.20131038
